# Angiotensin II type 1 receptor localizes at the blood–bile barrier in humans and pigs

**DOI:** 10.1007/s00418-022-02087-z

**Published:** 2022-02-28

**Authors:** Galyna Pryymachuk, Ehab El-Awaad, Nadin Piekarek, Uta Drebber, Alexandra C. Maul, Juergen Hescheler, Andreas Wodarz, Gabriele Pfitzer, Wolfram F. Neiss, Markus Pietsch, Mechthild M. Schroeter

**Affiliations:** 1 Department of Anatomy I, University of Cologne, Faculty of Medicine and University Hospital Cologne, Kerpener Str. 62, 50937 Cologne, Germany; 2 Institute II of Pharmacology, Center of Pharmacology, University of Cologne, Faculty of Medicine and University Hospital Cologne, Gleueler Str. 24, 50931 Cologne, Germany; 3 Department of Pharmacology, Faculty of Medicine, Assiut University, Assiut, 71515 Egypt; 4 Institute of Pathology, University of Cologne, Faculty of Medicine and University Hospital Cologne, Kerpener Str. 62, 50937 Cologne, Germany; 5 Experimental Medicine, University of Cologne, Faculty of Medicine and University Hospital Cologne, Ostmerheimer Str. 200, 51109 Cologne, Germany; 6 Institute for Neurophysiology, Center for Physiology and Pathophysiology, University of Cologne, Faculty of Medicine and University Hospital Cologne, Robert-Koch-Str. 39, 50931 Cologne, Germany; 7 Cologne Excellence Cluster Cellular Stress Response in Aging-Associated Diseases (CECAD), University of Cologne, Joseph-Stelzmann-Str. 26, 50931 Cologne, Germany; 8 Center for Molecular Medicine Cologne, University of Cologne, Robert-Koch-Str. 21, 50931 Cologne, Germany; 9 Institute of Vegetative Physiology, Center for Physiology and Pathophysiology, University of Cologne, Faculty of Medicine and University Hospital Cologne, Robert-Koch-Str. 39, 50931 Cologne, Germany

**Keywords:** AT1R, Human liver, Porcine liver, Canals of Hering, Gallbladder, Tight junctions

## Abstract

Animal models and clinical studies suggest an influence of angiotensin II (AngII) on the pathogenesis of liver diseases via the renin–angiotensin system. AngII application increases portal blood pressure, reduces bile flow, and increases permeability of liver tight junctions. Establishing the subcellular localization of angiotensin II receptor type 1 (AT1R), the main AngII receptor, helps to understand the effects of AngII on the liver. We localized AT1R in situ in human and porcine liver and porcine gallbladder by immunohistochemistry. In order to do so, we characterized commercial anti-AT1R antibodies regarding their capability to recognize heterologous human AT1R in immunocytochemistry and on western blots, and to detect AT1R using overlap studies and AT1R-specific blocking peptides. In hepatocytes and canals of Hering, AT1R displayed a tram-track-like distribution, while in cholangiocytes AT1R appeared in a honeycomb-like pattern; i.e., in liver epithelia, AT1R showed an equivalent distribution to that in the apical junctional network, which seals bile canaliculi and bile ducts along the blood–bile barrier. In intrahepatic blood vessels, AT1R was most prominent in the tunica media. We confirmed AT1R localization in situ to the plasma membrane domain, particularly between tight and adherens junctions in both human and porcine hepatocytes, cholangiocytes, and gallbladder epithelial cells using different anti-AT1R antibodies. Localization of AT1R at the junctional complex could explain previously reported AngII effects and predestines AT1R as a transmitter of tight junction permeability.

**Supplementary Information:**

The online version contains supplementary material available at 10.1007/s00418-022-02087-z.

## Introduction

The membrane-bound angiotensin II receptor type 1 (AT1R) is an abundant prototypical G-protein coupled receptor (GPCR) that is involved in many physiological and pathological pathways, including the liver. Previous research postulates the presence of two axes for the renin–angiotensin system (RAS) in the liver. The first, the classical axis, includes angiotensin II (AngII), the product of angiotensin-converting enzyme (ACE), which mediates the biological function through its main effector AT1R. In the second axis, Ang-(1–7), the product of ACE2, binds to the Mas receptor.

Upon binding of AngII to AT1R, this receptor mediates several important systemic effects in the liver, including vascular, proliferative, and inflammatory reactions (Shim et al. 2018). In dogs, intraportal infusion of AngII causes a strong increase of portal pressure followed by constriction of the intrahepatic portal ramifications (Scholtholt and Shiraishi 1968). In rats, AngII injection in the jugular vein produces a rapid increase in the arterial and portal venous pressure, a reduced bile flow and excretion rate (Bianciotti et al. 1994), and increases the permeability of tight junctions (TJ) in hepatocytes (Lowe et al. 1988).

AT1R in the liver was originally identified by radio- and fluorescent-labeled ligand binding assays. Eventually, AT1R expression was shown in resident liver parenchyma cells using different methods (Paxton et al. 1993; Leung et al. 2003; Campanile et al. 1982; Gasc et al. 1994; Schulte et al. 2009; Sen et al. 1983; Bataller et al. 2000). In humans and animal models, both AngII and AT1R are involved in the pathogenesis of nonalcoholic fatty liver disease (Li et al. 2019; Sturzeneker et al. 2019). In rat, bile duct ligation leads to upregulation of AT1R, especially in fibrotic areas (Paizis et al. 2002); while in healthy, quiescent hepatic stellate cells (HSCs), the components of the RAS are sparse and in the case of AngII not present at all. In cirrhotic liver, expression of angiotensinogen, ACE, and AngII is upregulated and the effects of AngII seem to be bidirectional (Bataller et al. 2003).

Although there is common knowledge about AT1R function in the biliary tree (hepatocytes, cholangiocytes, and gallbladder epithelial cells) (Patel 2003; Afroze et al. 2015; Campanile et al. 1982), little is known about the localization of the receptor. The membrane protein AT1R has also been detected in nuclei of hepatocytes (Schulte et al. 2009), but its subplasma membrane localization in situ has not been established.

In this study, we investigated the plasma membrane localization of AT1R in human and porcine liver, focusing on hepatocytes, cholangiocytes, and porcine gallbladder epithelial cells (GBECs). For this, we employed six different anti-AT1R antibodies that were directed towards various epitopes of the receptor. At first glance, staining of AT1R produced a tram-track-like pattern in hepatocytes. This pattern continued in the canals of Hering that are bordered by hepatocytes. In the sections of the canals of Hering that are formed by cholangiocytes, AT1R exhibited a honeycomb-like distribution. More detailed overlay studies indicated a localization of the plasma membrane-bound AT1R to the apical junctional complexes, where AT1R localized closer to the TJ (ZO-1, claudin-1, and symplekin) than to the adherens junctions (AJ) (E-cadherin) in hepatocytes and cholangiocytes.

## Material and methods

### Tissue preparation

Human liver tissue samples from the marginal border of resected liver specimens were collected from three patients with their informed and written consent according to the Biomasota code (13-091) at the University Hospital of Cologne. The study was approved by the ethics committee of the University Hospital of Cologne (18-052). Specimens of normal porcine liver and gallbladder (*n* = 5) were obtained from the Centre for Experimental Medicine at the University Hospital of Cologne. Age-matched samples were collected from 8- to 10-week-old healthy female Landrace × Pietrain hybrid pigs that were used to conduct a study unrelated to liver (Schroeder et al. 2017). The present study was approved by the governmental animal care and use committee (LANUV, North Rhine-Westphalia, Germany, 84-02.04.2014.A081 and 84-02.04.2014.A157). The entire livers, including gallbladders, were removed immediately post-euthanasia and randomly selected organ parts were trimmed to 1 cm^3^ blocks and snap-frozen in isopentane, pre-cooled with liquid nitrogen. The unfixed tissue blocks were embedded in Tissue-Tek^®^ O.C.T.™ compound (Sakura Finetek, Netherlands). Cryosections of 5–20 µm thickness were cut at –20 °C (SLEE Cryostat, Germany), thaw-mounted on chrome-gelatin-coated glass slides, and air-dried for 60 min at 37 °C.

### Antibodies

Commercial anti-AT1R antibodies raised against different epitopes of AT1R are listed in Supplementary Table 1. We established anti-AT1R-C18 (Santa Cruz, sc-31181, batch no. D0615) as reference antibody in the following study. It is worthwhile to mention that from the employed anti-AT1R antibodies listed in Supplementary Table 1, beside anti-AT1R-C18, only anti-AT1R-G3 was able to give an unambiguous signal of AT1R in immunohistochemistry (IHC), immunocytochemistry (ICC), and western blots. Further primary and fluorescence- or HRP-conjugated secondary antibodies used in this study are listed in Supplementary Tables 2 and 3.

### Immunofluorescence histochemistry

To establish the optimal reaction conditions for the anti-AT1R antibodies, air-dried unfixed cryosections (see earlier), as well as heterologous human AT1R (hhAT1R)-expressing HEK293-EBNA cells and sham-transfected control cells (Invitrogen, Carlsbad, CA, USA, passage no. 20) were used untreated. Alternatively, they were subjected to (a) immersion for 5 min in ice-cold acetone, methanol, or acetone-methanol mixture, followed by air-drying at room temperature (RT) for 60 min and rehydration with phosphate-buffered saline (PBS) for 10 min; or (b) fixation for 10 min with either 2% or 4% paraformaldehyde (PFA) at RT and washing with PBS, three times for 10 min each. Representative images of unfixed, acetone-treated, or alternatively fixed tissues are shown in Supplementary Fig. 1. For all specimens, the subsequent procedure was identical. Glass-mounted cryosections were permeabilized by 0.1% Triton X-100 plus 0.05% Tween-20 in PBS for either 15 min (tissues) or 5 min (cells) and incubated in blocking buffer (5% normal donkey serum (Dako) plus 0.05% Tween-20 in PBS) for 60 min at RT followed by overnight incubation with primary antibodies at 4 °C. After specimens were washed with PBS, containing 0.05% Tween-20 (three times for 5 min each), they were incubated with fluorescence-labeled secondary antibodies in the presence of 4′,6-diamidino-2-phenylindole (DAPI, 1 µg/mL, ThermoScientific) for 1 h at RT. For visualization of F-actin, Phalloidin-iFluor 488 (1:200, Abcam) was added to the secondary antibody mixture. Primary and secondary antibodies were diluted in antibody buffer (1% normal donkey serum plus 0.05% Tween-20 in PBS). For secondary antibody controls, cryosections or HEK293-EBNA cells were treated as described earlier but incubated with secondary antibodies only. All incubation steps were performed in a humidified chamber. After incubation with secondary antibody, specimens were washed once with PBS, containing 0.05% Tween-20 (5 min), then twice with PBS 5 min each, mounted with ProlongGold (ThermoScientific), and examined by confocal or wide-field fluorescence microscopy within 48 h. Except for the permeabilization, all other buffers used on human liver cryosections contained protease inhibitor cocktail (one tablet per 50 mL PBS, cOmplete™ ULTRA Tablets, Mini, EDTA-free, Roche).

### Microscopy

Depending on the availability of the confocal microscopes, images from liver and gallbladder cryosections, as well as from HEK293 cells, were acquired on the following laser-scanning confocal microscopes: Leica DMI 6000B (software LAS AF Version 2.6.0 build 7266, Leica Microsystems CMS GmbH, Germany) and Zeiss LSM880 (software Zen 3.2, version 3.2.0.0000, Carl Zeiss Microscopy GmbH, Germany). The confocal microscopes were equipped with the following objectives: HCX PL APO lambda blue 63.0 × 1.40 OIL UV or HCX PL APO CS 100.0 × 1.40 OIL (Leica DMI 6000B) and Plan-Apochromat 63 × /1.4 Oil DIC M27 (Zeiss LSM880). Depending on the fluorochromes, the specimens were illuminated by a 405-nm diode UV laser for DAPI, an argon laser for Alexa Fluor 488, a DPSS 561 laser (Leica DMI 6000B) or a DPSS 561-10 laser (Zeiss LSM880) for Alexa Fluor 568, and a HeNe 633 laser for Alexa Fluor 647. To eliminate possible cross-excitation between different fluorescent labels, confocal images were recorded in sequential scan mode at RT. The device specific software was used for adjustment of signal intensity and generation of both maximum intensity projections (MIP) and Z-stack images. All figures were composed using Adobe Photoshop CS3 Extended 10.0 software version 22.5.1 (Adobe Systems, USA). Linear adjustments in contrast and brightness of Fig. 3 were applied to the entire images of panels a–c. Further details in regard to the acquisition of images are provided in Supplementary Table 4.

### Miscellaneous methods

Detailed information about plasmid construction for heterologous expression of hhAT1R, cell culture, ICC, preparation of hhAT1R in whole cell lysates and enriched plasma membrane fractions, SDS-PAGE, western blotting, and specificity controls of primary and secondary antibodies are given in the Supplementary information.

## Results

### Premise

The goal of the present study was to elucidate the subcellular localization of AT1R in liver and gallbladder epithelial cells in situ. To overcome the restricted availability of human tissue and to confirm our findings, we also determined the localization of AT1R in porcine liver and gallbladder sections. In porcine liver, we found the identical membrane-bound AT1R distribution as in human tissue.

The reported results are valid under the assumption that the detected AT1R signals are authentic. Our detailed experiments to identify and validate antibodies that were able to detect AT1R in human and porcine tissues, as well as in hhAT1R-expressing HEK293-EBNA cells, are represented in the Supplementary information.

It is most important to stress that the presented results were gained under optimized fixation methods, i.e., air-dried cryosections of unfixed tissue that were acetone-treated and permeabilized with Triton X-100 plus Tween-20. For a detailed description of establishing these conditions, see Supplementary information. PFA fixation or treatment with methanol or methanol/acetone (1:1) severely impeded localization and intensity of anti-AT1R immunostaining (for details see Supplementary Fig. 1). Furthermore, note that identical localization of AT1R was observed with both confocal microscopes.

## Localization of AT1R

**Fig. 1:**
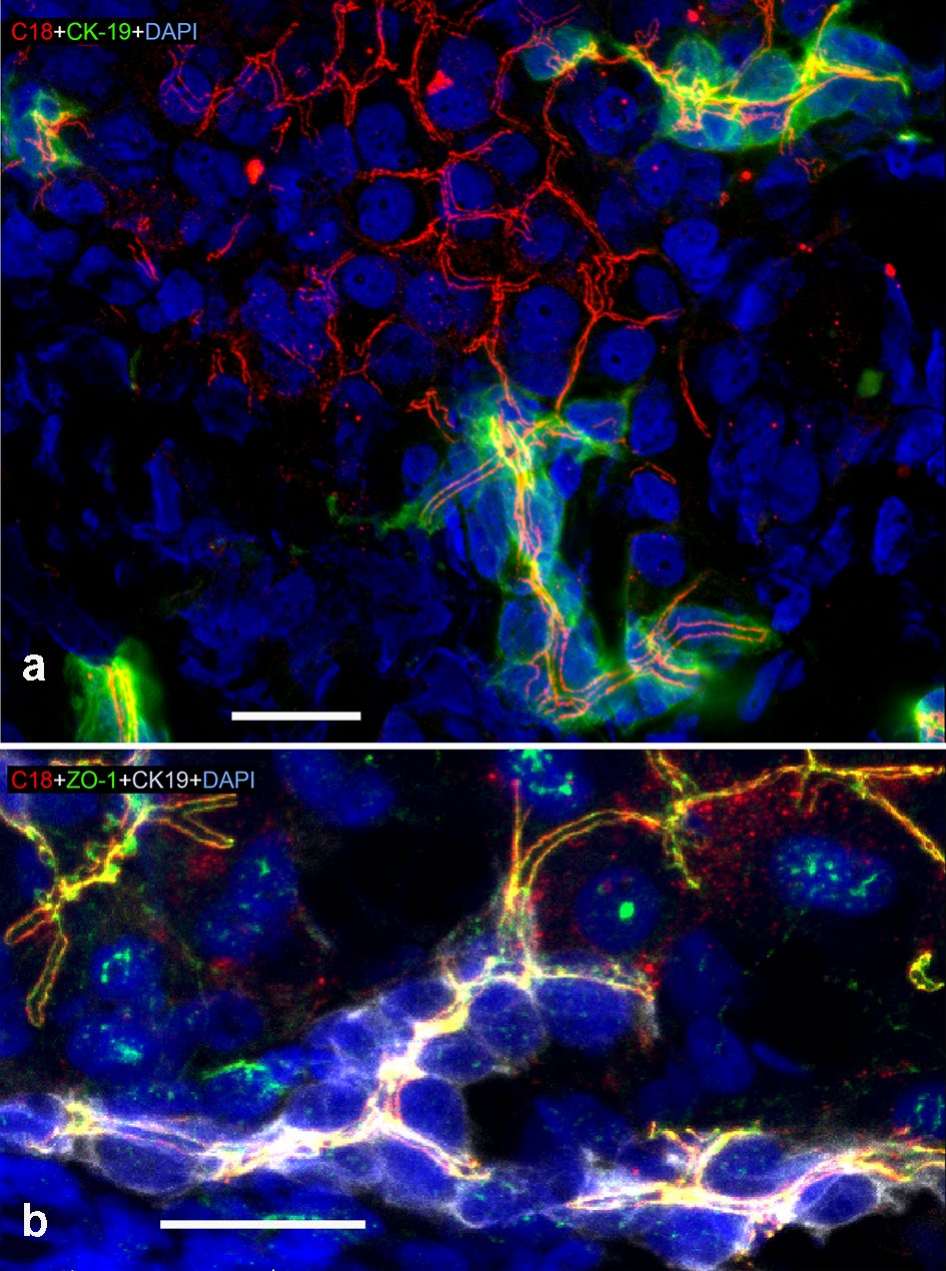
Distribution of AT1R in porcine (**a**) and human (**b**) liver. Liver cryosections were incubated with anti-AT1R-C18 (red), anti-CK-19 (green (**a**) or gray (**b**)), DAPI (blue, nuclei), and in (**b**), additionally, with anti-ZO-1 (green). CK-19 indicated the presence of cholangiocytes. Where hepatocyte plates formed bile canaliculi, AT1R appeared as tram-track-like pattern which, similar to ZO-1, continued in the canal of Hering. Partial overlap of anti-AT1R and anti-ZO-1 signals are shown in yellow (**b**). Confocal microscopes **a** Zeiss LSM880; **b** Leica DMI 6000B. Scale bars 20 µm (**a**, **b**)

In hepatocytes—as a new and remarkable observation—AT1R showed up as tram-track-like pattern (Fig. 1). This AT1R immunoreactivity showed the classical staining pattern of bile canaliculi. The AT1R distribution was very similar to the localization of the tight junctional proteins represented by ZO-1 (Fig. 1b), typical for hepatocytes (Mensa et al. 2011; Keon et al. 1996; Anderson et al. 1989). However, we did not observe AT1R in the liver sinusoidal endothelium with the methods applied in this study. In the liver lobules, hepatocytes were further distinguished by their big round nuclei and—on the basis of the distance between the majority of adjacent nuclei—accordingly large cell volumes, their ability to form plates and trabeculae, and their surrounding environment, which provided no remarkable luminal space. While mature hepatocytes do not express cytokeratin 19 (CK-19) (van Eyken et al. 1987), as shown in Fig. 1, CK-19 was used as a typical marker for cholangiocytes by us and others (Pryymachuk et al. 2013; Tanimizu et al. 2021; Yuan et al. 2018).

The canals of Hering are found near the outer edge of the classic liver lobule and function as the connection between the bile canaliculi and interlobular bile ducts (Fig. 1). The canals of Hering are bordered in their origin by hepatocytes, where we found AT1R localized in a tram-track-like pattern. Later on, before the canals lead into bile ducts, they are lined by the CK-19-expressing cholangiocytes and oval cells (Saxena and Theise 2004).

**Fig. 2:**
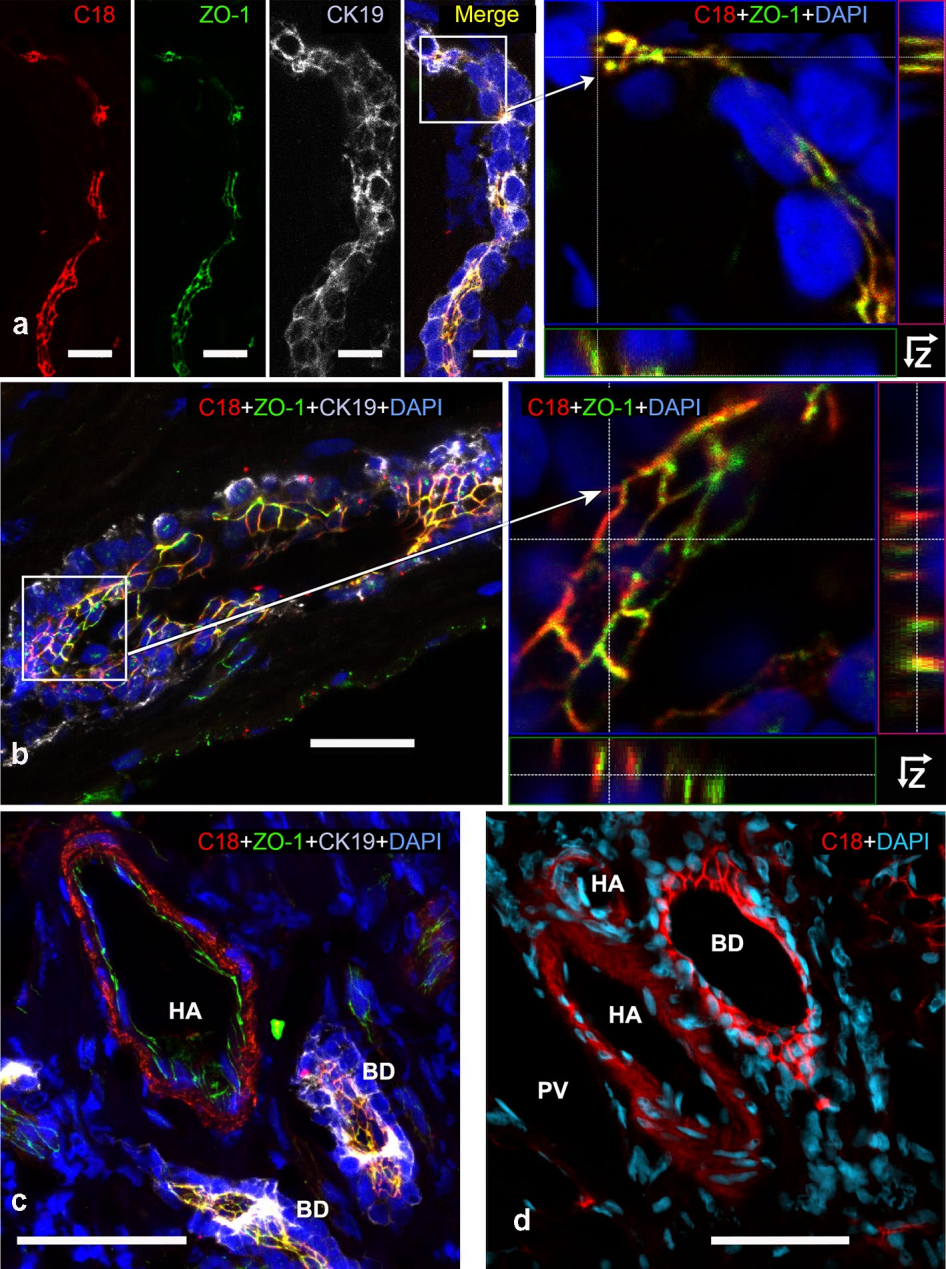
AT1R localization in the human (**a**–**c**) and porcine (**d**) intrahepatic biliary tree. Liver cryosections incubated with anti-AT1R-C18 (red), and DAPI (blue, nuclei) (**a**–**d**), and additionally with anti-ZO-1 (green, TJ), anti-CK-19 (gray, cholangiocytes) in (**a**–**c**); partial signal overlap (anti-AT1R and anti-ZO-1) in yellow. AT1R and ZO-1 localize towards the apical membrane of cholangiocytes (**a**–**c**). Small (**a**) and larger (**b**–**d**) bile ducts are shown. *HA* hepatic artery, *BD* bile duct. (**a**, left), (**b**, left), **c** and **d** are shown as MIP; (**a**, right) and (**b**, right) are Z-stack analyses. Confocal microscopes **a**–**c** Leica DMI 6000B; **d** Zeiss LSM880. Scale bars 20 µm (**a**, **b**), 50 µm (**c**, **d**)

In cholangiocytes, AT1R followed the honeycomb-like pattern (Fig. 2a) of the apical junctional complex of polarized epithelial cells (Grosse et al. 2012; Keon et al. 1996). With increasing canal diameter, the honeycomb-prototype became more prominent (Fig. 2a, b).

Intrahepatic bile ducts within the portal area presented a fully developed honeycomb-like pattern when probed with anti-AT1R-C18 (Fig. 2b–d). Intrahepatic bile ducts were identified by the CK-19-positive cholangiocytes that surrounded their lumen in a single layer (Figs. 2b, c). Cholangiocytes were further distinguished from other hepatic epithelial cells by their prismatic morphology and basally located round to oval nuclei (Fig. 2d). Junctional complexes connect the cholangiocytes among each other. Incubating oblique bile duct sections with appropriate antibodies directed against junctional proteins resulted in a honeycomb-like pattern as well (Fig. 2a–c, Supplementary Fig. 2).

In blood vessels within the portal area, AT1R, as expected, was present in branches of the hepatic artery, as shown before (Wang et al. 2015). In human and pig tissues, AT1R was mainly found in smooth muscle cells within the tunica media, in major branches of the hepatic artery, and small arteries of the portal area (Fig. 2c, d, Supplementary Fig. 3a–c). AT1R was also found—albeit to a much lower degree—in endothelial cells of the tunica intima marked with CD31 (Supplementary Fig. 3a, b, d).

**Fig. 3:**
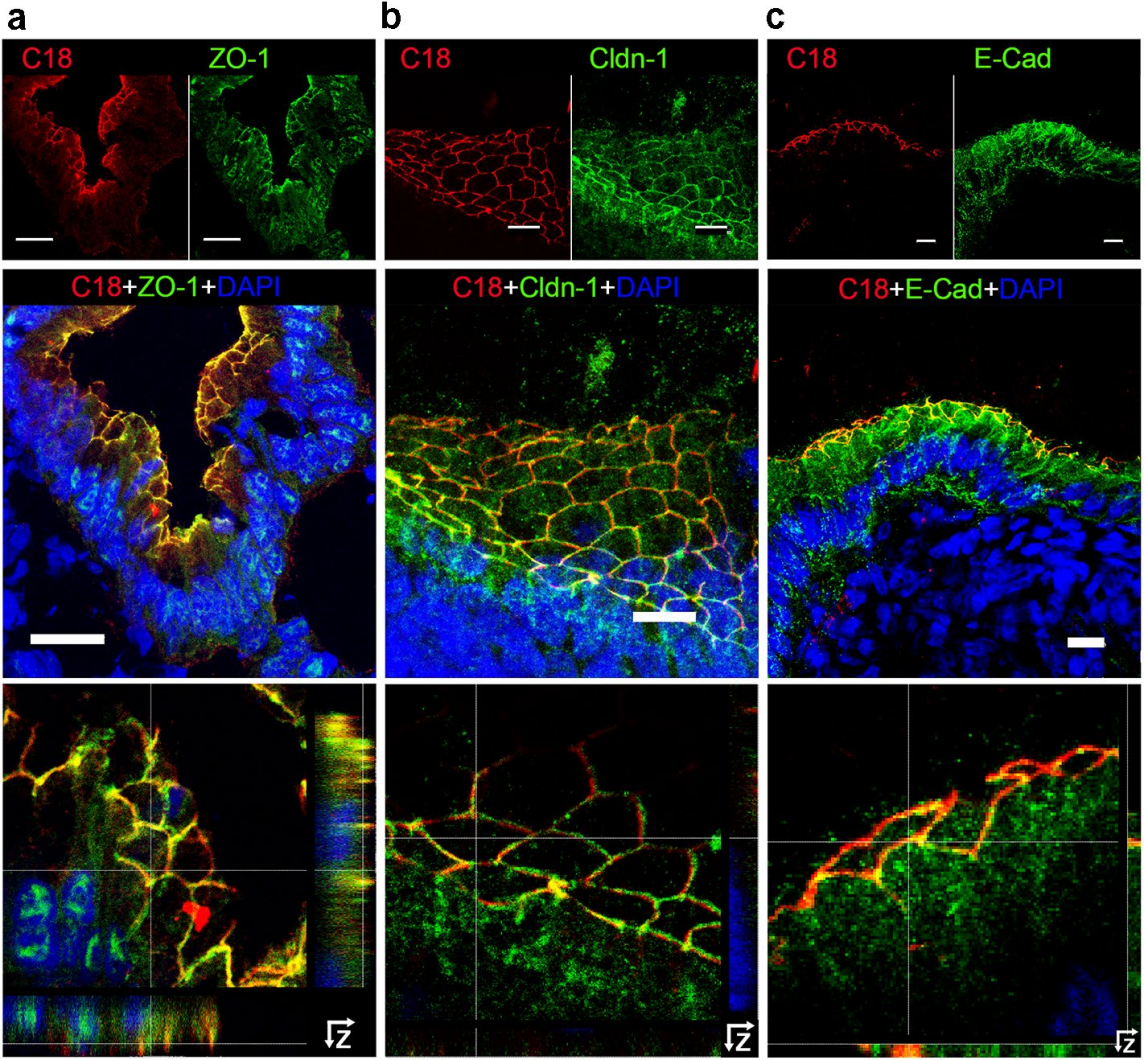
AT1R colocalizes in gallbladder epithelial cells with TJ proteins ZO-1 and claudin-1 and closely associates with AJ protein E-cadherin. Porcine gallbladder cryosections were incubated with anti-AT1R-C18 (red) (**a**–**c**), anti-ZO-1 (**a**), anti-claudin-1 (Cldn-1) (**b**), and anti-E-cadherin (E-Cad) (**c**) (all in green), (top row). Nuclei were stained with DAPI (blue). Merged images (in the middle row) show colocalization of AT1R with either ZO-1 or claudin-1 (yellow). Top and middle rows: MIP; bottom row: enlarged regions of merged images above with Z-stack projections at the right side and below the images. Confocal microscope Leica DMI 6000B. Scale bars 20 µm (**a**), 10 µm (**b**, **c**)

In the gallbladder, high prismatic CK-19-positive cells, i.e., GBECs, line the lumen and their oval nuclei are basally located (Supplementary Fig. 4). AT1R followed the characteristic honeycomb-like structure described for the junctional complex (Fig. 3). Our results suggest a localization of AT1R in the biliary tree within or in close vicinity to the junctional complex.

## Colocalization of AT1R with proteins of the junctional complex

In the plasma membrane of the biliary tree, symplekin, claudin-1, and ZO-1 are used as marker proteins for TJ (Keon et al. 1996; Nemeth et al. 2009). E-cadherin is enriched in AJ (Nemeth et al. 2009), whereas desmoglein 2 is specific for desmosomes (Zhou et al. 2015). We determined the localization of AT1R with respect to these proteins using double-labeling IHC.

**Fig. 4:**
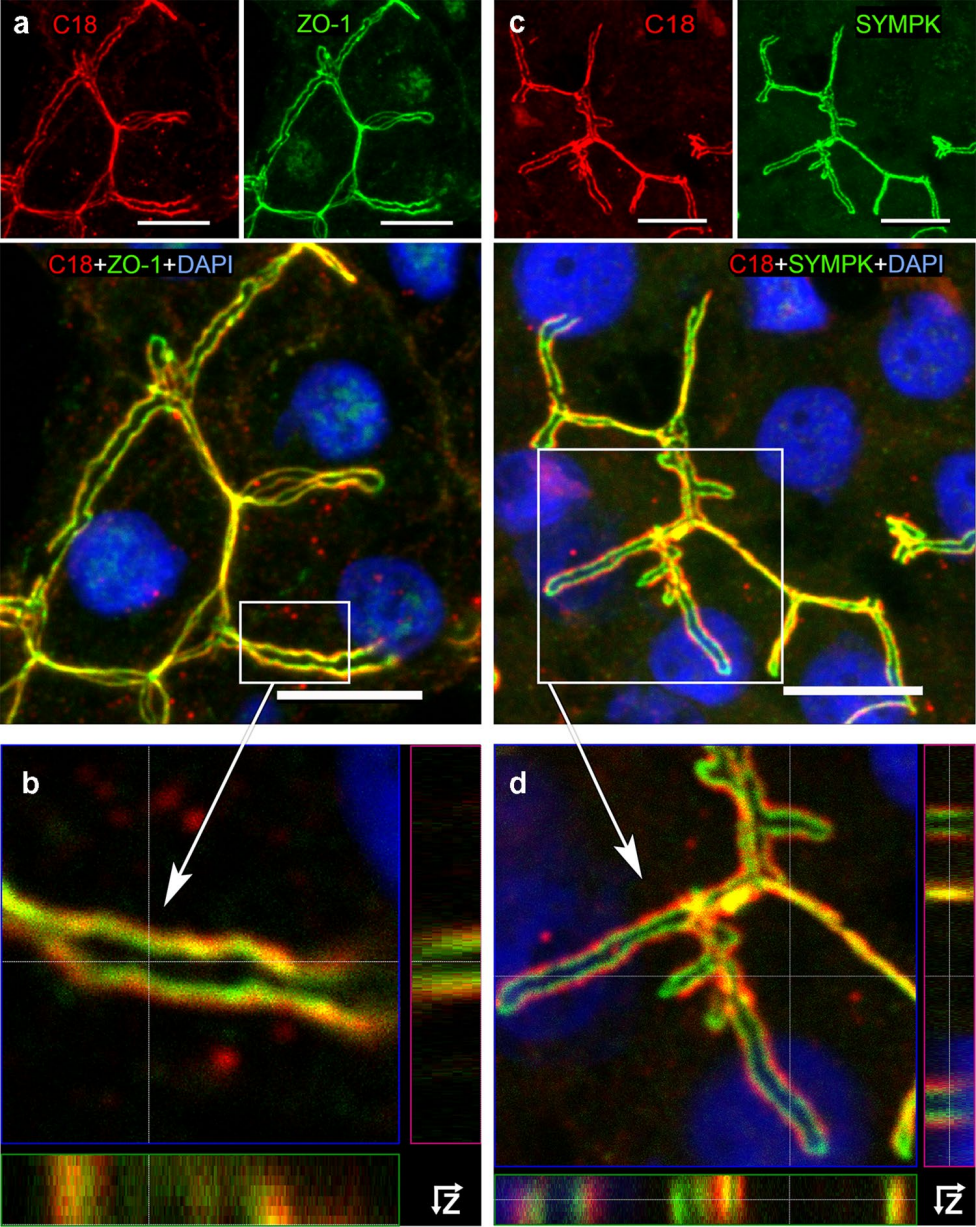
AT1R colocalizes in human hepatocytes with the TJ proteins ZO-1 and symplekin (SYMPK). Liver cryosections incubated with anti-AT1R-C18 (red) and anti-ZO-1 (green) in **a** or with anti-symplekin (green) in **c** are shown in separate fluorescence channels (top panels). Merged MIP images (bottom panels in **a** and **c**) include DAPI (blue, nuclei) and show the overlap of AT1R with the TJ signals (ZO-1 (**a**) and symplekin (**c**)) in yellow. **b** and **d** show enlarged views of the boxed regions from **a** and **c** with Z-stack analyses. Confocal microscope Leica DMI 6000B. Scale bars 10 µm

In human hepatocytes, longitudinal sections of bile canaliculi (BC) showed a partial overlapping appearance of AT1R (red) and ZO-1 (green), forming parallel lines (Fig. 4a). Z-stacking, where the BC were presented as cross sections (Fig. 4b), revealed that two parallel double-punctual arrangements were hidden behind the double lines. While AT1R and ZO-1 partially overlapped (yellow), ZO-1 appeared to be luminally (apically) oriented, bordering the lumen of BC between two neighboring hepatocytes (Anderson et al. 1989); AT1R, however, seemed to locate to the lateral membrane space.

In colocalization studies of AT1R (red) and symplekin (green), (Fig. 4c, Z-stacks in Fig. 4d) a similar pattern with partial overlap was observed. The green symplekin signal was mainly found on the luminal side. Overlap of the AT1R and symplekin signals are shown in yellow.

**Fig. 5:**
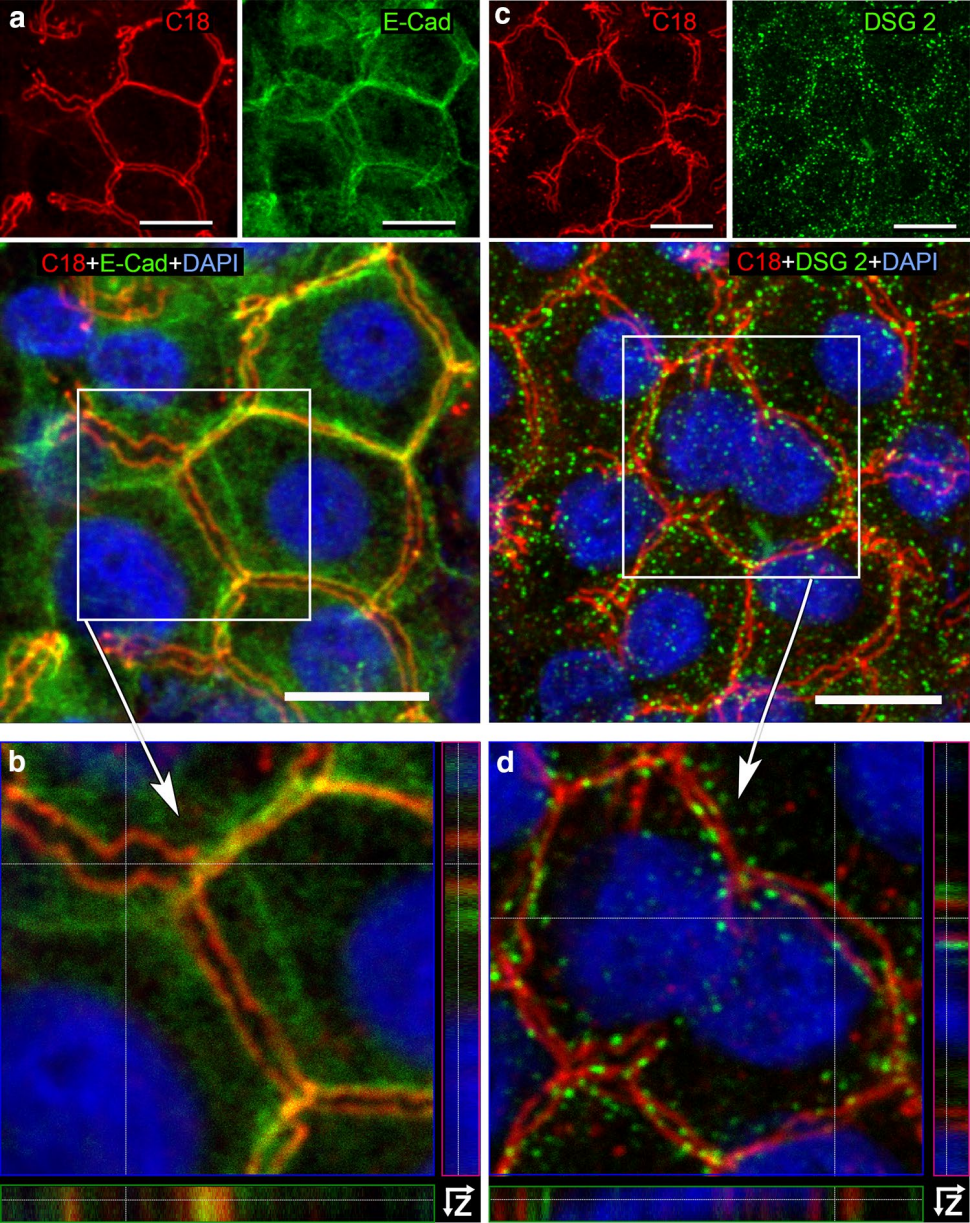
In human hepatocytes, AT1R is adjacent to adherens junctions and desmosomes but does not show systematic overlap with these structures. Liver cryosections were incubated with anti-AT1R-C18 (red) (**a**–**d**), anti-E-cadherin (E-Cad, green) (**a**, **b**), or anti-desmoglein 2 (DSG 2, green) (**c**, **d**) and DAPI (blue, nuclei). In **a** and **c** the specimens are shown in separate fluorescence channels (top panel), merged MIP images (bottom panel). Detailed images of boxed regions from **a** and **c** are shown with Z-stack analyses in **b** and **d**, respectively. Confocal microscope Leica DMI 6000B. Scale bars 10 µm

E-cadherin represents the AJ, which appeared here as broad ribbon-like structures with different densities (Fig. 5a). The highest E-cadherin concentration was observed close to the canalicular membrane domain. Compared to TJ, AT1R (red) and E-cadherin (green) proteins appeared in AJ in reverse order, namely from luminal (AT1R) to basolateral (E-cadherin), see Z-stacks in Fig. 5b. The AT1R tracks (red) were punctually framed by the desmosomal protein desmoglein 2 (green), see Fig. 5c, Z-stacks in Fig. 5d. Depending on the angle of view, we could observe a sporadic colocalization of AT1R with desmosomes (yellow).

**Fig. 6:**
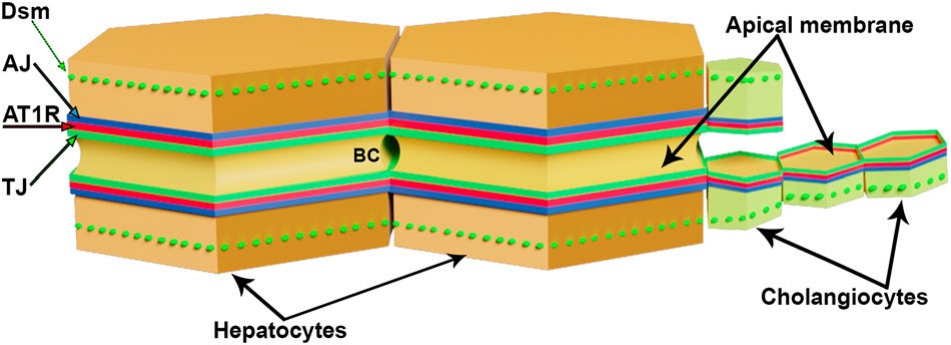
Proposed model of the organization of AT1R within the junctional complex in hepatocytes and cholangiocytes. TJ, tight junctional marker proteins (green); AT1R (red); AJ, adherent junctional marker protein (blue); Dsm, desmosomal marker protein (green dotted line); BC, bile canaliculus

Porcine hepatocytes (Supplementary Fig. 5) presented the same colocalization pattern of AT1R with TJ proteins [ZO-1 and claudin-1 (green)] as observed in human. Both TJ proteins circumvented the BC lumen and overlapped partially (yellow) with AT1R (red), placing AT1R in close proximity to TJ. In Z-stacks ZO-1 and claudin-1 signals were luminal oriented, whereas AT1R tended to the cytoplasmic side. Note, AT1R was never detected in-between the tracks of TJ proteins in both human and porcine tissues (e.g., Fig. 4 and Supplementary Fig. 5). No localization of AT1R to the apical plasma membrane of hepatocytes was found. In brief, these colocalization studies suggest that in hepatocytes the AT1R is situated exactly at the borderline between the lumen of the bile canaliculi and the intercellular space between hepatocytes which leads into the space of Disse and the blood sinus of the liver. More specifically—as far as light microscopy can resolve this question—AT1R appears to be situated at the basolateral plasma membrane of hepatocytes, as shown in Fig. 6.

In intrahepatic bile ducts, the AT1R-ZO-1 pattern continued and generated a honeycomb-like structure. In both longitudinal and oblique views, it became more visible as the small BDs merged into large ducts (Fig. 2b, c and Supplementary Fig. 2). Z-stacks of cholangiocytes (Fig. 2a, b) show a clear luminal orientation of ZO-1 (green) and basolateral directed AT1R (red). Both proteins overlapped considerably (yellow), underlining the close vicinity of AT1R to TJ, placing ZO-1 closer to the duct lumen than AT1R.

In GBECs, AT1R was found between TJ and AJ (Fig. 3a–c) in proximity to the apical-oriented TJ proteins ZO-1 or claudin-1 and partially overlapped with them. AT1R was also in close vicinity to the basolateral-oriented AJ protein E-cadherin. All junctional complex proteins displayed a honeycomb-like structure. In GBECs, we observed the same arrangement as earlier in hepatocytes and cholangiocytes (Fig. 6), following the pattern TJ–AT1R–AJ.

## Discussion

The goal of the present study was to explore the membrane-bound localization of AT1R in normal human liver and gallbladder in situ. As far as investigations were restricted by the availability of human tissue, such as gallbladder sections, porcine material was employed to support and complement the research. We observed endogenous AT1R localized at the basolateral membrane of hepatocytes and lateral membrane of cholangiocytes in human and porcine liver and in porcine GBECs. Colocalization studies with proteins of the junctional complex showed AT1R in immediate vicinity to TJ, as indicated by a high overlap with the TJ proteins ZO-1, claudin-1, and symplekin. To a much lower degree, an overlap of AT1R with the adherens junctional protein E-cadherin was observed. Sporadically, a colocalization of AT1R with the desmosomal protein desmoglein 2 was seen. The arrangement of AT1R within the junctional complex is summarized in Fig. 6. Results were identical in all investigated samples of human liver and likewise in all investigated samples of porcine liver.

As binding of AngII to AT1R leads to upregulation of factors contributing to inflammation and fibrogenesis, exact knowledge of receptor localization is a prerequisite for therapeutic strategies. Receptor localization influences strength and specificity of cellular signalling (Itzhak et al. 2016; Hung and Link 2011) and therefore organ function. Despite extensive studies concerning the localization of AT1R in other organs, our current knowledge regarding its subcellular localization in the liver is based largely on functional assays (Booz et al. 1992). An increasing body of evidence states AT1R as ultimate target of the classical RAS (Simoes et al. 2017). Physiological and pathophysiological effects of circulating AngII on the liver and gallbladder are transmitted by downstream signalling pathways (Munshi et al. 2011; Zong et al. 2015). Blockade of AT1R was explored in animal models and in clinical studies in order to develop therapeutic strategies to reduce liver fibrosis (Munshi et al. 2011) and cholestasis (Patel 2003; Paizis et al. 2002).

Earlier studies, set out to examine AT1R in rat, mice, or human liver, were designed to determine AGTR1 expression and AT1R protein synthesis depending on the stage of liver disorder such as fibrosis, cirrhosis, cholestasis, or NAFDL (Schulte et al. 2009; Paizis et al. 2002; Afroze et al. 2015; Leung et al. 2003). In previous studies, the incidence of AT1R in the liver was postulated on the basis of northern blot analysis, RT-PCR (Bataller et al. 2003), ligand binding (Bataller et al. 2000), in situ hybridization (Gasc et al. 1994), and autoradiography assays (Paizis et al. 2002). In contrast to these methods, IHC procedures offer the opportunity to visualize AT1R at subcellular resolution (Fonseca and Brown 1997). To the best of our knowledge, membrane-bound AT1R has not been shown before on subcellular level in liver tissue. Sporadically, we observed cytoplasmic and nuclear-associated localization of AT1R. Previously, AT1R has been associated with the nucleus (Schulte et al. 2009; Booz et al. 1992). As AT1R is considered to be a membrane-bound GPCR activated by circulating AngII, we aimed to determine its exact localization in the plasma membrane of human and porcine liver and gallbladder.

AT1R has been described as abundant receptor in vascular smooth muscle cells, responsible for the mediation of vasoconstriction (Dasgupta and Zhang 2011). In agreement with the literature (Gunther et al. 1982), we found AT1R localized to smooth muscle cells of the tunica media in branches of the hepatic artery. We refrained from examining AT1R in blood vessels on subcellular level; instead, we focused on its localization in the biliary tree. AT1R in smooth muscle cells was merely used as anatomical control. It should be noted that we found endothelial cells of the hepatic artery to be AT1R-positive, too. AT1R presence in endothelial cells has been described before (Khayat et al. 2018).

Canalicular bile, secreted by adjacent hepatocytes, flows in biliary capillaries, passes the canals of Hering, and enters intrahepatic and finally extrahepatic bile ducts to be stored in the gallbladder. Through all these routes, bile is surrounded by semipermeable barriers of TJ proteins, i.e., the so-called blood–bile barrier (Kojima et al. 2003), and thus is separated from the pericellular environment.

In hepatocytes, cholangiocytes, and GBECs, localization of AT1R was not completely identical to that of the tight junctional proteins (ZO-1, claudin-1, or symplekin); however, the signal overlap of these proteins with the AT1R signal suggested very close vicinity. Remarkably, AT1R was enclosed by basolateral located AJs, represented by E-cadherin. Thus, AT1R localized in hepatocytes mainly at the lateral plasma membrane domain located between TJ and AJ, as summarized in Fig. 6. In cholangiocytes and GBECs, the junctional complex seals the lateral intercellular space of neighboring cells only close to the apical plasma membrane. Here again, AT1R was found as part of the junctional complex, positioned between TJ and AJ (Fig. 6). Nascent AT1R traffics to the plasma membrane via the microtubule network (Zhang et al. 2013). In hepatocytes, microtubules form a dense network below the BCs, which interacts with actin (Novikoff et al. 1996). BCs are surrounded by two circumferential actin belts. The first actin belt regulates vesicle flow close to the plasma membrane; the second actin belt is formed by actomyosin fibers containing short filamentous actin (F-actin) and myosin II (Tsukada and Phillips 1993). The F-actin crisscrosses with the perpendicularly running microtubule network (Novikoff et al. 1996). In hepatocytes, F-actin showed a subcortical distribution, which was most prominent at the canalicular plasma membrane, where F-actin was lumen-oriented. Towards basolateral points of contact, the close vicinity of F-actin and AT1R suggests a colocalization of the two proteins (Supplementary Fig. 6). The myosin II of the actomyosin fibers is associated with AJs and is responsible for BC-contractility (Tsukada and Phillips 1993). AngII-induced stimulation of AT1R results in an actomyosin-mediated contractile response in epithelial cells (Cuerrier et al. 2009), contributing to bile flow (Tsukada and Phillips 1993).

The observed physical localization of AT1R enables this receptor to fulfil its physiological function as GPCR. Binding of AngII to the membrane-anchored N-terminus of AT1R induces the G protein-mediated C-terminal receptor–effector interaction (Touyz and Schiffrin 2000; Shirai et al. 1995; de Almeida et al. 1994). Alternatively, AT1R can be activated by physiological membrane stretch (Durvasula et al. 2004; Ramkhelawon et al. 2013; Mederos y Schnitzler et al. 2011; De Mello 2012). The AT1R interacts with Gα subunits, such as Gαq/11, Gα12/13, and Gαi (St-Pierre et al. 2018; Shatanawi et al. 2011). AT1R coupled to Gαq/11 works as cell surface mechanosensor in cardiomyocytes and in smooth muscle cells of small renal and cerebral resistance arteries (Mederos y Schnitzler et al. 2011). Furthermore, AT1R was established as mechanosensor that stimulates proliferation of cultured rat cholangiocytes (Munshi et al. 2010; Afroze et al. 2015). In hepatocytes, and probably other liver cells (Kim et al. 2018; Wang et al. 2000), Gα12 and Gαi2 localize to the TJ region where they bind to a GPCR (Dodane and Kachar 1996; Meyer et al. 2002).

Just as cholangiocytes, hepatocytes are mechano-sensitively regulated (Burton et al. 2020). In hepatocytes and cholangiocytes we found AT1R localized between TJs and AJs, a predestined position to work as mechanosensor. Remarkably, in hepatocytes increased paracellular permeability can be induced by the interaction of Gα12 with ZO-1 via the tyrosine kinase Src (Meyer et al. 2002). Perfusion of liver with AngII also increased paracellular permeability and is considered to reflect a specific receptor-mediated hormonal effect (Lowe et al. 1988). The herein determined morphological localization of AT1R is supported by previously performed functional measurements (Lowe et al. 1988).

In conclusion, our morphological studies of human and porcine liver in combination with the reported functional evidence that AngII increases TJ permeability (Lowe et al. 1988) point to AT1R as the regulating receptor for TJ permeability in hepatocytes, cholangiocytes, and GBECs. The proposed localization of AT1R between TJ and AJ in bile route-forming cells suggests that AT1R may function as linker and mediator between TJ and AJ activity in the biliary tree.

## Supplementary Information

Below is the link to the electronic supplementary material.

Supplementary file1 (PDF 2693 kb)

## Data Availability

The data sets generated during and/or analyzed during the current study are available from the corresponding author on reasonable request.
