# Supplementary material for: Angiotensin II type 1 receptor localizes at the blood–bile barrier in humans and pigs
Source: Histochem Cell Biol. 2022 Feb 28;157(5):513–24. doi: 10.1007/s00418-022-02087-z (PMC9114028; doi:10.1007/s00418-022-02087-z)
Supplement: Supplementary file 1 — Supplementary file1 (PDF 2693 kb) [file 418_2022_2087_MOESM1_ESM.pdf]

Angiotensin-II type1 receptor localizes at the blood–bile barrier in humans and pigs

Histochemistry and Cell Biology

Pryymachuk, Galyna; El-Awaad, Ehab; Piekarek, Nadin; Drebber, Uta; Maul, Alexandra C.; Wodarz, Andreas; Pfitzer, Gabriele; Neiss, Wolfram F.; Pietsch, Markus; Schroeter, Mechthild M.

Corresponding author: Galyna Pryymachuk, Department of Anatomy I, University of Cologne, Faculty of Medicine and University Hospital Cologne, Kerpener Str. 62, D-50937, Cologne, Germany

Phone: +49 221 478-5653, Fax: +49 221 478-87691, Email: galyna.pryymachuk@uk-koeln.de

ORCID identifier: 0000-0002-6971-2802 (https://orcid.org/0000-0002-6971-2802)

Supplementary information content

Supplementary Methods **3**

Results and Discussion **5**

Supplementary Table 1. List of used anti-AT1R antibodies. **12**

Supplementary Table 2. List of primary antibodies other than anti-AT1R. **13**

Supplementary Table 3. List of secondary antibodies. **15**

Supplementary Table 4. Image acquisition information. **16**

Supplementary Figures and Legends **25**

Supplementary Fig 1 AT1R-recognition by anti-AT1R-C18 antibody in differently pre-treated human liver cryosections **25**

Supplementary Fig 2 AT1R distribution in porcine bile ducts **27**

Supplementary Fig 3 AT1R localizes predominantly in smooth muscle cells of hepatic artery **29**

Supplementary Fig 4 AT1R localizes to GBECs **31**

Supplementary Fig 5 AT1R does not localize in-between the tracks of TJ proteins **32**

Supplementary Fig. 6 AT1R locates in close vicinity to F-actin at the apical site of human and porcine hepatocytes **33**

Supplementary Fig 7 Anti-AT1R-C18 and anti-AT1R-G3 antibodies produce congruent tram-track-like pattern in hepatocytes 34

Supplementary Fig 8 Tram-track-like distribution of AT1R in human hepatocytes detected by anti-AT1R-C18 antibody in comparison to detection by other anti-AT1R antibodies 35

Supplementary Fig 9 Anti-AT1R-C18, anti-AT1R-G3 and anti-Strep-tag® II antibodies provided an identical detection pattern of hhAT1R in HEK293-EBNA cells 37

Supplementary Fig 10 Anti-AT1R-ab59018 and anti-Strep-tag® II antibodies produced congruent hhAT1R-signals in HEK293-EBNA cells 38

Supplementary Fig 11 Signal loss of anti-AT1R-C18 antibody after pre-adsorption with AT1R-specific blocking peptide 39

Supplementary Fig 12 Signal loss after blocking of both anti-AT1R-C18 and anti-AT1R-G3 antibodies by an AT1R-specific peptide 40

Supplementary Fig 13 MALDI-TOF fingerprint analysis locates blocking peptide to the C-terminus of human and porcine AT1R 43

Supplementary Fig 14 Detection of hhAT1R in Western blots 44

Supplementary Methods

Plasmid construction. The human wild-type full-length AT1R cDNA (accession No. NM_000685; cDNA Resource Center (www.cdna.org), CloneID: AGTR100000, catalogue no. AGTR10TN01) was subcloned into a modified episomal expression vector pCEP-Pu (kindly provided by Prof. Dr. Manuel Koch, Center for Biochemistry and Institute for Dental Research and Oral Musculoskeletal Biology, University of Cologne), after introduction of the restriction sites NheI and XhoI. The same restriction sites were used for cloning. The vector backbone contained a carboxyl-terminal thrombin cleavage site and a double Strep-tag® II sequence in-frame with N-terminal BM-40 signal peptide (Gara et al. 2008). The construct pCEP-Pu-AGTR1 was confirmed by sequencing (Eurofins Genomics Germany GmbH).

Cell culture and heterologous human AT1R. HEK293-EBNA cells (Invitrogen, Carlsbad, CA, USA, passage no. 20) were grown in Dulbecco's Modified Eagle's Medium (DMEM) with high glucose (Gibco) supplemented with 10% fetal calf serum (SIGMA), 100 U/mL penicillin, 100µg/mL streptomycin (Gibco), and L-glutamine (2mM/L, Gibco). Cells were cultured at 37°C in a humidified atmosphere with 5% CO_2. Cells were transfected with pCEP-Pu-AGTR1 (transfected cells = T) or empty pCEP-Pu (control cells = C) (0.25–0.5µg/525cm2) when 80-90% confluent, using TurboFect transfection reagent (Thermo Fischer Scientific, Germany). Puromycin selection (3µg/mL) was started 24h post transfection. Cells were kept under continuous selection pressure and split when 80-90% confluent.

For immunocytochemistry, cells expressing heterologous human AT1R (hhAT1R) or control cells were seeded on glass coverslips and cultured in selection medium. Coverslips were carefully removed when cells were 60-80% confluent, 3h air-dried at RT, and stored at -20°C.

Pre-adsorption with blocking peptide. For pre-adsorption studies, a commercial blocking peptide (sc-31181P, Lot C2814, Santa Cruz Biotechnology Inc., USA) was used according to the manufacturer’s instructions with an antibody to blocking peptide ratio of 1:1 (w/w).

Cryosections of human and porcine liver were incubated with anti-AT1R-C18 antibody with or without pre-adsorption with blocking peptide. Specimens of hhAT1R-expressing HEK293-EBNA cells were similarly incubated side-by-side either with anti-AT1R-C18 or anti-AT1R-G3 antibodies or using the same antibodies pre-adsorbed with blocking peptide. The sequence of the blocking peptide was identified by mass spectrometry (Central Bioanalytics, CMMC, Cologne, Germany).

Preparation of whole cells lysates and plasma membrane. Whole cell lysates were obtained by a modified method according to Sharma et al. (Sharma et al. 2012). In brief, 80-90% confluent hhAT1R-expressing HEK293-EBNA or control cells were mechanically harvested and immediately transferred into conical tubes. Cells were centrifuged (200RCF, 5min at 4°C) and cell pellets were suspended in ice cold PBS. After repeating the washing step twice, the resulting pellets were lysed in ice cold lysis buffer (10mM Tris, 1mM EDTA, 1% SDS, 0.1% Triton X-100, and 1mM phenylmethylsulfonyl fluoride, 200µl/10^6 cells) supplemented with cOmplete (Roche) and stored on ice for 60min. Subsequently, the lysate was centrifuged at 20,800RCF for 60min at 4°C and the supernatants were aliquoted, immediately frozen and stored at -80°C until use. For enrichment of the plasma membrane the method of Lund et al. was employed (Lund et al. 2009). Briefly, the cells were mechanically harvested as before. About 1.4×10^8 cells were transferred into conical tubes and centrifuged (200RCF, 5min at 4°C). Pellets were suspended in pre-cooled PBS and centrifuged. This washing step was repeated twice. Subsequently cell pellets were washed with 1.5mL hypotonic buffer (HB) (10mM Tris-HCl pH 6.8, 1.5mM $MgCl_2$ and 10mM NaCl) and immediately centrifuged. Finally, cell pellets were suspended in 3mL HB, incubated for 5min on ice, and centrifuged for 5min at 310RCF, 4°C. Cell pellets were suspended in 1.5mL lysis buffer (255mM sucrose, 20mM HEPES pH 7.4, 1mM EDTA supplemented with cOmplete (Roche)) and 3min homogenized on ice using a motor-driven potter (500rpm). The homogenates were centrifuged for 10min at 20,080RCF at 4°C to remove cell debris. Supernatants were transferred into

ultracentrifuge tubes and centrifuged for 200min in a TLA-55 rotor (Beckman) at 54,000rpm at 4°C. Supernatants were carefully removed and pellets were dissolved in 50µL PBS including cOmplete (Roche), frozen in liquid N2 and stored at -20°C. Tissue samples were directly homogenized in 1× Laemmli buffer containing cOmplete protease inhibitor cocktail (Roche). Protein concentration of the samples was estimated by Bradford using BSA as standard.

SDS-PAGE and Western blotting. Proteins were mixed with 2× Laemmli sample buffer containing 1% SDS and 50mM dithiothreitol. Alternatively, the membrane pellets were homogenized in 6M urea, 1% SDS, and 10mM TCEP. In both cases, reaction mixtures were incubated for 10min at RT. Increasing protein amounts were separated on 10% SDS-acrylamide gels. After electrophoresis, the proteins were transferred onto nitrocellulose-membranes, 0.2µm (#88024, Thermo ScientificTM) using Tobwin transfer buffer (192mM glycine, 25mM Tris, 0.01% SDS, and 10% methanol). Blots were blocked for 30min at RT with 5% (w/v) skimmed milk powder in PBS containing 0.1% Tween-20 and incubated overnight at 4°C with primary antibody. The following day, blots were incubated with the appropriate horseradish peroxidase-conjugated secondary antibodies for 1h. Protein bands were visualized with PierceTM ECL Western Blotting Substrate (#32106, Thermo ScientificTM) by exposure of the immunoblots to Amersham HyperfilmTM ECL (#28906835, GE Healthcare, Germany). Quantification of protein bands was performed by densitometric scans using the software Phoretix 1D (Version 5.00) with Rubber Band algorithm for background subtraction (Biostep, Germany).

Results and Discussion

Effect of treatment and fixatives of liver cryo-sections on recognition of AT1R by anti-AT1R-C18 antibody. In air-dried, Triton-X100-permeabilized, untreated or acetone-treated cryosections of fresh-frozen, i. e. unfixed liver tissue, anti-AT1R-C18 antibody detected AT1R in plasma membranes of hepatocytes with a tram-track-like appearance (Supplementary Fig.

1a). Treatment with methanol or methanol/acetone (1:1) produced a fuzzy signal non-related to morphological structures, probably due to loss of epitope recognition. Aldehyde fixation of the cryosections for 10 minutes at room temperature with 2% in PBS prior to incubation with anti-AT1RC18 antibody resulted in an aggregation-like appearance of the signals with scarcely visible tram-track-like patterns. Increasing the PFA concentration to 4% caused further aggregation of anti-AT1R-C18 with concomitant reduction of tram-track-associated signal.

As expected (Mardones and Gonzalez 2003), in air-dried, Triton-X100-permeabilized cryosections of unfixed tissue that were otherwise untreated prior to immunocytochemical incubation, no redistribution of membrane proteins took place, but the drawback was a low preservation of morphological structures. Acetone, as organic solvent and coagulative fixative, removed lipids, caused cytoplasmic flocculation, and tissue shrinkage. The latter is a well-known problem of most fixatives (Tran et al. 2015; Noguchi et al. 1997). However, acetone instantaneously stabilizes the cell membrane and precipitates proteins to their cellular architecture by dehydration (Hughes and Jones 2011; Hall et al. 1987; Bhattacharyya et al. 2010; Horobin 1982). Consequently, acetone precipitated AT1R with its seven transmembrane-spanning domains to the plasma membrane and retained it in the cell compartment without redistribution. Whereas acetone did not preserve morphological structures as PFA does, it preserved a plethora of epitopes that are destroyed by PFA fixation (Noguchi et al. 1997). In sections fixed with 2% PFA, anti-AT1R-C18 was able to recognize mainly membrane-associated epitopes and revealed only seldom track-like structures. Loss of immunoreactivity after PFA fixation is attributed to formation of inter- and intramolecular methylene bridges between the amino groups of proteins (Scalia et al. 2017). PFA fixation has been reported to induce blebs in plasma membrane (Fox et al. 1985), and hence is responsible for morphological artifacts. We therefore refrained from using tissues fixed with PFA or methanol, as no antigen-antibody interactions or insufficient interactions were observed.

Specificity of secondary antibodies and autofluorescence. For evaluation of the antibodies in human and porcine liver sections, we determined the specificity of the secondary fluorescent antibodies and autofluorescence of the investigated specimens. This is exemplary shown for human and porcine liver cryosections and secondary anti-goat Alexa®568 antibody in Supplementary Fig. 1b, 11a and 11d. With exception of the internal elastic membrane of arteries (Supplementary Fig. 3a), neither specific secondary antibody signals nor autofluorescence were observed under the chosen settings.

Detection of AT1R by anti-AT1R antibodies. Previous studies found several antibodies from commercial sources to be unspecific and giving false positive signals (Herrera et al. 2013a; Benicky et al. 2012; Rateri et al. 2011; Herrera et al. 2013b; Michel et al. 2009; Bouressam et al. 2018). Due to the ongoing debate concerning the specificity of anti-AT1R antibodies, we first singled out antibodies that were capable of specifically detecting AT1R by immunohistochemistry (IHC) and immunocytochemistry (ICC) (Supplementary Fig. 7-12) (Lorincz and Nusser 2008; Fritschy 2008). Initially, we employed six anti-AT1R antibodies to corroborate the localization of AT1R (Supplementary Table 1). With these antibodies we aimed not only to detect the C-terminus of AT1R, but also the N-terminus and/or the central region of the protein. However, plasma membrane-associated AT1R localization was only confirmed with three anti-AT1R antibodies directed against C-terminal epitopes of the receptor (anti-AT1R-C18, anti-AT1R-G3, and anti-AT1R-(306) sc-579 (Supplementary Fig. 7 and 8), with the former two antibodies giving signals of tram-track-like appearance in liver cryosections. An additional nucleus-associated AT1R appearance was suggested by two anti-AT1R antibodies, i.e. anti-AT1R-(306) sc-579 (Supplementary Fig. 8a) and anti-AT1R-NBP1-70997 (Supplementary Fig. 8b). In all cases, increasing antibody concentrations or prolonged incubation resulted in higher background (data not shown). Note of worth, the performance of anti-AT1R-G3 depended strongly on the lot number, resulting in a high background.

Investigating the five antibodies (anti-AT1R-C18, anti-AT1R-G3, anti-AT1R-(306) sc-579, anti-AT1R-ab59018 and anti-AT1R-NBP1-70997) on hhAT1R-expressing HEK293-EBNA cells, only anti-AT1R-C18 and anti-AT1R-G3 produced congruent clear signals with complete colocalization (Supplementary Fig. 9 and 10). The recognition of hhAT1R was further confirmed by simultaneous incubation of either anti-AT1R-C18 or anti-AT1R-ab59018 with anti-Strep-tag® II antibody (Supplementary Fig. 9c and 10b respectively). As anti-AT1R-G3 antibody and anti-Strep-tag® II antibody were both raised in mice, co-incubation was not performed. For the same reason, simultaneous incubation of anti-AT1R-C18 and anti-AT1R-ab59018 (both raised in goats) was omitted.

Taken together, the used antibodies performed optimal in air-dried cryosections, treated with ice-cold acetone and permeabilized with Triton X-100 plus Tween-20. Worth of note, when sections were fixed with PFA, antibodies performed poorly as shown for anti-AT1R-C18 (Supplementary Fig. 1). This might be a reason why other authors described problems, while trying to detect AT1R (Rateri et al. 2011). On the other hand, acetone-treated cryosections are often successfully employed for localization studies in liver and gall bladder (Keon et al. 1996; Anderson et al. 1989; Aust et al. 2004). In our investigations, anti-AT1R-C18 was the superior antibody in both *in situ* and *in vitro* studies and was therefore employed when we set out to determine the localization of AT1R.

Identification of AT1R-expressing cells. Histological structures and cell types were identified by features of the DAPI-stained nuclei, such as shape, distribution pattern, and their appearance to luminal space. Cholangiocytes, GBEC, and vascular endothelial cells were further identified by specific antibodies, i.e. anti-CK-19 antibody and anti-CD31 antibody, respectively.

Blocking Peptide. Pre-adsorption of anti-AT1R-C18 with a commercial blocking peptide (1:1, w/w) caused almost complete signal loss in human and porcine hepatocytes (Supplementary Fig. 11) and in hhAT1R-expressing HEK293-EBNA cells (Supplementary Fig. 12a). Signal

loss was also observed when hhAT1R-expressing HEK293-EBNA cells were incubated with identically pre-adsorbed anti-AT1R-G3 antibody (Supplementary Fig. 12b). Determination of the blocking peptide sequence by MALDI-TOF (m/z) fingerprint analysis resulted in the core-peptide KYIPPKAKSHS (Supplementary Fig. 13a). This highly conserved polypeptide is located within the last 50 amino acids of the carboxyl terminus of AT1R and is 100% identical with the human and porcine AT1R sequence (Supplementary Fig. 13b). This led to the conclusion that both the anti-AT1R-C18 and the anti-AT1R-G3 antibodies are directed against the C-terminus of AT1R and that the antibodies detect an AT1R-specific motif.

Detection of heterologous human AT1R protein by Western blotting. Based on the anti-AT1R antibody validation *in situ*, we employed anti-AT1R-C18 and anti-AT1R-G3 antibodies for detection of AT1R by Western blotting. The specificity of anti-AT1R antibodies was determined in electrophoretically separated total cell lysates from hhAT1R-expressing HEK293-EBNA (T) and empty vector-transfected control (C) cells by Western blotting (Supplementary Fig. 14a, 14b, left). The used anti-AT1R-C18 and anti-AT1R-G3 antibodies detected unambiguous bands at the expected molecular mass of hhAT1R monomers (42kDa). The prominent band at a molecular mass approximately double that of the hhAT1R monomer (84kDa), which has been observed before by Barki-Harrington *et al.* (Barki-Harrington et al. 2003) (Fig. 5a in that work, lower immunoblot, lane 8) suggests the presence of a AT1R homodimer. Homodimerization of heterologous AT1R has previously been shown (Hansen et al. 2004; Hansen et al. 2009; Young et al. 2017). Increased protein load was associated with a signal increase of these bands. Faint bands on the level of AT1R in control lysates suggested the presence of endogenous receptor protein as confirmed by RT-PCR, data not shown. Secondary antibody controls (Supplementary Fig. 14a and 14b, right) produced faint bands at about 60-65kDa.

AT1R is well-known as integral plasma membrane receptor. We subjected transfected HEK293-EBNA cells to subcellular fractioning and enriched hhAT1R. The enriched plasma membranes were analyzed by Western blotting and incubated with anti-AT1R-C18 and anti-AT1R-G3 antibodies as well as with the anti-Strep-tag® II antibody (Supplementary Fig. 14c). Here, all primary antibodies also detected protein bands corresponding to AT1R monomers and potential homodimers. The additionally performed secondary antibody controls (Supplementary Fig. 14c) did not result in protein bands, as found for total cell lysates. The monoclonal anti-AT1R-G3 antibody detected an additional weak band of unknown nature in hhAT1R-expressing HEK293-EBNA cells at a molecular mass of about 45kDa.

Both antibodies anti-AT1R-C18 and anti-AT1R-G3 were able to detect hhAT1R in cell lysates and in membrane-enriched fractions. Again, these results were backed up by an anti-Strep-tag® II antibody (Supplementary Fig. 14c). However, AT1R was only detected when a minimum of 30µg total protein was loaded. The loaded target protein concentration and the species-specific primary amino acid sequence determine the recognition by an antibody. The main differences of our study to previous reports were both the protein amount loaded (Herrera et al. 2013a) and the investigated species (Herrera et al. 2013a; Rateri et al. 2011). In these reports, low protein amounts from total tissue or cell lysates were loaded. In addition, these groups aimed to detect rodent AT1R whereas we investigated the human AT1R. Our results are in line with those of Sharma et al. (Sharma et al. 2012) who successfully detected hhAT1R loading 15µg total protein/slot.

In our hands, anti-AT1R-antibodies failed to detect AT1R in Western blots of human and porcine liver lysates, even when 200µg protein was loaded. A mass spectrometric analysis of SDS-PAGE-separated lysates (50µg protein) did not reveal the presence of AT1R either, which is most likely caused by the low amount of AT1R protein in the sample. Our results indicated

that although AT1R is an abundant receptor in plasma membranes, its amount in the lysates is so low, that it did not meet the antibodies' detection limits.

Supplementary Table 1. List of used anti-AT1R antibodies.

**Trade name/order number** (manufacturer)	Batch number	Citation	Host & Clonality	Epitope	Dilution		Signal****)		
					IHC/ICC	WB	IHC	ICC	WB
**ab59018** (Abcam PLC)	GR39459-7	(Tang et al. 2018)	Go**), pol***)	C-terminal	1:30	1:750	?	+	ND
**AT1R (N-10)/ sc-1173** (Santa Cruz*)	G2115	(Herrera et al. 2013a; Sharma et al. 2012)	Rb, pol	N-terminal	1:30	1:500	?	-	-
**AT1 (C-18)/ sc-31181** (Santa Cruz)	D0615	(Rateri et al. 2011)	Go, pol	C-terminal	1:70	1:500	+	+	+
**AT1(G-3)/ sc-515884** (Santa Cruz)	B0119	(Macedo et al. 2021)	M, mo	C-terminal	1:50	1:700	+	+	+
**AT1 (306)/ sc-579** (Santa Cruz)	A1410	(Benicky et al. 2012)	Rb, pol	C-terminal	1:30	1:500	+	-	ND
**NBP1-70997** (Novus Biologicals)	61212		Rb, pol	Central region	1:50	1:700	+	?	ND

*) Santa Cruz Biotechnology, Inc.; **) Go: goat; Rb: rabbit; M: mouse; ***) pol: polyclonal; mo: monoclonal; ****) +: positive signal, -: no signal, ?: questionable signal, ND: not determined.

Supplementary Table 2. List of primary antibodies other than anti-AT1R.

**Trade name/order number** (manufacturer)	Batch number	Citation	Host & Clonality	Epitope	Dilution	
					IHC	WB
**NovocastraTM Cytokeratin 19 (clone B170)** NCL-L-CK19/CK19-L-U (Leica Biosystems)	6006479	(Fujita et al. 2000)	M*), mo**)	human cytokeratin 19 intermediate filament protein	1:30-1:60	ND ***)
**ZO-1 Polyclonal Antibody (ZMD.437)/40-2300** (Thermo Fisher Scientific/ Invitrogen)	1117635A	(Helm et al. 2020; Penes et al. 2005)	Rb, pol	synthetic peptide derived from the N-terminal region of human, dog, mouse, and rat ZO-1	1:25	ND
**Claudin 1 Antibody (MH25)/ 71-7800** (Thermo Fisher Scientific/ Invitrogen)	1215331A	(Shin et al. 2020)	Rb, pol	synthetic peptide derived from the C-terminus of human/mouse claudin-1 protein	1:25	ND
**anti-Desmoglein 2/ 610121** (PROGEN Biotechnik GmbH)	302061	(Schafer et al. 1996)	Rb, pol	recombinant peptide (extracellular repeat domain E2 of human desmoglein 2	1:70-1:100	ND

Supplementary Table 2 continued. List of primary antibodies other than anti-AT1R.

**Trade name/order number** (manufacturer)	Batch number	Manufacturer	Host & Clonality	Epitope	Dilution	
					IHC	WB
**anti-Symplekin (Sym-TJ-E150)/651100** (PROGEN Biotechnik GmbH)	1487	(Keon et al. 1996)	M, mo	FTKVVLEAPLITESALE, corresponding to AA532-548 of human symplekin (Keon et al. 1996)	1:30	ND
**E-Cadherin (24E10)/ 3195** (Cell Signaling Technology, Inc.**)**	10	(Schneider et al. 2014)	Rb, mo	synthetic peptide corresponding to the sequence surrounding Pro780 of human E-cadherin protein	1:30	ND
**Anti-CD31/ ab28364** (Abcam PLC)	6R229032-1	(Jung et al. 2021)	Rb, pol	synthetic peptide within murine CD31 aa 700 to the C-terminus (C terminal)	1:50	ND
**StrepMAB-Classic/ 2-1507-001 (**IBA GmbH**)**	1507-0042	(Durvasula et al. 2004)	M, mo	Strep-tag®II and Twin-Strep-tag®	1:50	1:1000
**anti-GAPDH [EPR1689]/ ab181602** (Abcam PLC)	GR217575-9 GR194633-12	(Guo et al. 2021)	Rb, mo	recombinant fragment within mouse GAPDH aa 100 to the C-terminus (UniProtKB - P16858)	ND	1:20,000

*) Rb: rabbit; M: mouse; **) pol: polyclonal; mo: monoclonal; ***) ND: not determined.

Supplementary Table 3. List of secondary antibodies.

Trade name/order number	Batch number	Manufacturer	Host & Clonality	Dilution	
				IHC/ICC	WB
anti-mouse IgG (H+L)-Alexa Fluor® 488/A-21202	1022448	Life Technologies	Do*), pol**)	1:300	ND ***)
anti-mouse IgG (H+L)-Alexa Fluor® 568/A-10037	989784	Life Technologies	Do, pol	1:300	ND
anti-goat IgG (H+L)-Alexa Fluor® 568/A-11057	1010042	Life Technologies	Do, pol	1:300	ND
anti-goat IgG (H+L)-Alexa Fluor® 647/A-21447	1010075	Life Technologies	Do, pol	1:300	ND
anti-rabbit IgG (H+L)-Alexa Fluor® 488/A-21206	1028736	Life Technologies	Do, pol	1:300	ND
anti-rabbit/HRP (P0217)/P021702-2	20007702	Dako	pig, pol	ND	1:4000
anti-mouse/HRP (P0260)/P026002-2	20066043	Dako	Rb, pol	ND	1:1000
anti-mouse IgG H&L (HRP)/ab97023	GR3313784-1	Abcam	Go, pol	ND	1:1000
anti-goat IgG (HRP)/A5420	064M477-8	Sigma	Rb, pol	ND	1:2000

*) Do: Donkey; Rb: rabbit; Go: goat; **) pol: polyclonal; ***) ND: not determined.

Supplementary Table 4. Image acquisition information.

Fig.Nr	Confocal microscope	Objective	Detectors gain at excitation wavelength				Pixel	Scan-Direction	Absolute Time (Leica DMI 6000B)/ Frame Time (Zeiss LSM880)	image bit depth
			405 nm	488 nm	561 nm or 514 nm[a]	633 nm				
1a	Zeiss LSM880	Plan-Apochromat 63x/1.4 Oil DIC M27	474.0	776.0	653.0	-	2096×2096	1	3 min 54 s	12 bits
1b	Leica DMI 6000B	HCX PL APO lambda blue 63.0x1.40 OIL UV	717	810	56	1210	512×512	2	15 min	8 bits
2a	Leica DMI 6000B	HCX PL APO CS 100.0x1.40 OIL	547	810	56	1050	1024×1024	2	20 min 04 s	8 bits
2b	Leica DMI 6000B	HCX PL APO lambda blue 63.0x1.40 OIL UV	701	1195	31	797	2048×2048	2	29 min 48 s	8 bits

2c	Leica DMI 6000B	HCX PL APO lambda blue 63.0x1.40 OIL UV	613	1195	31	737	2048×2048	2	27 min 44 s	8 bits
2d	Zeiss LSM880	Plan-Apochromat 63x/1.4 Oil DIC M27	540.6	-	951.7	-	1976×1976	1	1 min 18 s	12 bits
3a middle	Leica DMI 6000B	HCX PL APO lambda blue 63.0x1.40 OIL UV	866	810	10	-	512×512	2	1 min 56 s	8 bits
3a bottom	Leica DMI 6000B	HCX PL APO lambda blue 63.0x1.40 OIL UV	866	810	10	-	512×512	2	2 min 37 s	8 bits
3b	Leica DMI 6000B	HCX PL APO CS 100.0x1.40 OIL	800	1077	800	-	512×512	2	2 min 33 s	8 bits
3c	Leica DMI 6000B	HCX PL APO CS 100.0x1.40 OIL	797	890	780	-	512×512	2	2 min 6 s	8 bits
4a, 4b	Leica DMI 6000B	HCX PL APO CS 100.0x1.40 OIL	744	1195	156	-	1024×1024	2	12 min 21 s	8 bits
4c, 4d	Leica DMI 6000B	HCX PL APO CS 100.0x1.40 OIL	657	1066	156	-	1024×1024	1	22 min 23 s	8 bits

5a, 5b	Leica DMI 6000B	HCX PL APO CS 100.0x1.40 OIL	661	868	156	-	1024×1024	2	8 min 7 s	8 bits
5c, 5d	Leica DMI 6000B	HCX PL APO CS 100.0x1.40 OIL	727	1195	156	-	1024×1024	2	9 min 16 s	8 bits
S1a unfixed	Zeiss LSM880	Plan-Apochromat 63x/1.4 Oil DIC M27	576.0	746.0	746.0	-	796×796	1	47.21 s	12 bits
S1a acetone	Zeiss LSM880	Plan-Apochromat 63x/1.4 Oil DIC M27	576.0	746.0	742.0	-	2048×2048	1	4 min 3 s	12 bits
S1a methanol	Zeiss LSM880	Plan-Apochromat 63x/1.4 Oil DIC M27	576.0	746.0	751.0	-	796×796	1	1 min 34 s	12 bits
S1a acetone/methanol	Zeiss LSM880	Plan-Apochromat 63x/1.4 Oil DIC M27	576.0	746.0	751.0	-	796×796	1	1 min 34 s	12 bits
S1a 2% PFA	Zeiss LSM880	Plan-Apochromat 63x/1.4 Oil DIC M27	576.0	746.0	751.0	-	796×796	1	1 min 34 s	12 bits

S1a 4% PFA	Zeiss LSM880	Plan-Apochromat 63x/1.4 Oil DIC M27	576.0	746.0	751.0	-	796×796	1	1 min 34 s	12 bits
S1b unfixed	Zeiss LSM880	Plan-Apochromat 63x/1.4 Oil DIC M27	576.0	746.0	746.0	-	796×796	1	1 min 34 s	12 bits
S1b acetone	Zeiss LSM880	Plan-Apochromat 63x/1.4 Oil DIC M27	576.0	746.0	753.0	-	796×796	1	47.21 s	12 bits
S1b methanol	Zeiss LSM880	Plan-Apochromat 63x/1.4 Oil DIC M27	576.0	746.0	751.0	-	796×796	1	1 min 34 s	12 bits
S1b acetone/methanol	Zeiss LSM880	Plan-Apochromat 63x/1.4 Oil DIC M27	576.0	746.0	751.0	-	796×796	1	1 min 34 s	12 bits
S1b 2% PFA	Zeiss LSM880	Plan-Apochromat 63x/1.4 Oil DIC M27	576.0	746.0	751.0	-	796×796	1	1 min 34 s	12 bits
S1b 4% PFA	Zeiss LSM880	Plan-Apochromat 63x/1.4 Oil DIC M27	576.0	746.0	751.0	-	796×796	1	1 min 34 s	12 bits

S2	Leica DMI 6000B	HCX PL APO CS 100.0x1.40 OIL	811	26	34	-	1024×1024	2	19 min 34 s	8 bits
S3	Leica DMI 6000B	HCX PL APO CS 100.0x1.40 OIL	722	990	80	-	1024×1024	2	18 min 55 s	8 bits
S4	Zeiss LSM880	Plan-Apochromat 63x/1.4 Oil DIC M27	541.8	706.0	809.0	-	1504×1504	1	1 min 29 s	12 bits
S5a	Leica DMI 6000B	HCX PL APO CS 100.0x1.40 OIL	848	26	143	-	512×512	1	19 min 38 s	8 bits
S5b	Leica DMI 6000B	HCX PL APO CS 40.0x1.25 OIL UV	832	105	79	-	512×512	1	5 min	8 bits
S6a	Zeiss LSM880	Plan-Apochromat 63x/1.4 Oil DIC M27	541,8	607,2	640,4	-	2048x2048	1	7,45 s	12 bits
S6b	Zeiss LSM880	Plan-Apochromat 63x/1.4 Oil DIC M27	541,8	607,2	640,4	-	1024×1024	1	20,26 s	12 bits
S6c	Zeiss LSM880	Plan-Apochromat 63x/1.4 Oil DIC M27	553,0	587,0	688,0	-	1708x1708	1	3 min 22 s	12 bits

S6d	Zeiss LSM880	Plan-Apochromat 63x/1.4 Oil DIC M27	553,0	587,0	688,0	-	684x684	1	46,56	12 bits
S7a	Leica DMI 6000B	HCX PL APO lambda blue 63.0x1.40 OIL UV	727	990	156	-	1024×1024	2	11 min 12 s	8 bits
S7b	Leica DMI 6000B	HCX PL APO lambda blue 63.0x1.40 OIL UV	727	990	156	-	1024×1024	2	11 min 12 s	8 bits
S7c	Zeiss LSM880	Plan-Apochromat 63x/1.4 Oil DIC M27	621.7	751.0	786.8	-	2264×2264	1	2 min 14 s	12 bits
S7d	Zeiss LSM880	Plan-Apochromat 63x/1.4 Oil DIC M27	621.7	751.0	786.8	-	664×664	1	6.58 s	12 bits
S8a	Leica DMI 6000B	HCX PL APO lambda blue 63.0x1.40 OIL UV	600	1195	156	-	512×512	2	2 min 43 s	8bits
S8b	Leica DMI 6000B	HCX PL APO lambda blue 63.0x1.40 OIL UV	698	1195	156	-	1024×1024	2	7 min 20 s	8 bits

S9a	Leica DMI 6000B	HCX PL APO CS 100.0x1.40 OIL	878	208	974[a]	-	512×512	2	4 min 54 s	8 bits
S9b	Leica DMI 6000B	HCX PL APO CS 100.0x1.40 OIL	878	208	974[a]	-	512×512	2	5 min 26 s	8 bits
S9c	Leica DMI 6000B	HCX PL APO lambda blue 63.0x1.40 OIL UV	878	53	32	-	512×512	2	12 min 26 s	8 bits
S10a	Leica DMI 6000B	HCX PL APO CS 100.0x1.40 OIL	1230	208	1139[a]	-	512×512	2	8 min 16 s	8 bits
S10b	Leica DMI 6000B	HCX PL APO CS 40.0x1.25 OIL UV	898	215	295	-	512×512	2	3 min 45 s	8 bits
S10c	Leica DMI 6000B	HCX PL APO lambda blue 63.0x1.40 OIL UV	703	990	80	-	1024×1024	2	8 min 6 s	8 bits
S11a	Leica DMI 6000B	HCX PL APO lambda blue 63.0x1.40 OIL UV	667	-	80	-	1024×1024	2	3 min 51 s	8 bits
S11b	Leica DMI 6000B	HCX PL APO lambda blue 63.0x1.40 OIL UV	659	-	80	-	1024×1024	2	5 min 9 s	8 bits

S11c	Leica DMI 6000B	HCX PL APO lambda blue 63.0x1.40 OIL UV	606	-	80	-	1024×1024	2	3 min 51 s	8 bits
S11d	Leica DMI 6000B	HCX PL APO lambda blue 63.0x1.40 OIL UV	719	-	80	-	1024×1024	2	2 min 35 s	8 bits
S11e	Leica DMI 6000B	HCX PL APO lambda blue 63.0x1.40 OIL UV	719	-	80	-	1024×1024	2	1 min 17 s	8 bits
S11f	Leica DMI 6000B	HCX PL APO lambda blue 63.0x1.40 OIL UV	696	-	80	-	1024×1024	2	1 min 17 s	8 bits
S12a top	Zeiss LSM880	Plan-Apochromat 63x/1.4 Oil DIC M27	491.1	-	716.0	-	2146×2146	1	21.13 s	12 bits
S12a middle	Zeiss LSM880	Plan-Apochromat 63x/1.4 Oil DIC M27	476.0	-	836.0	-	1451×1406	1	1 min 30 s	12 bits
S12a bottom	Zeiss LSM880	Plan-Apochromat 63x/1.4 Oil DIC M27	491.1	-	716.0	-	2146×2146	1	21.13 s	12 bits

S12b top	Zeiss LSM880	Plan-Apochromat 63x/1.4 Oil DIC M27	523.3	-	685.0	-	1306×1306	1	14.03 s	12 bits
S12b middle	Zeiss LSM880	Plan-Apochromat 63x/1.4 Oil DIC M27	579.0	-	685.0	-	1198×1198	1	1.21 s	12 bits
S12b bottom	Zeiss LSM880	Plan-Apochromat 63x/1.4 Oil DIC M27	571.8	-	685.0	-	1918×1918	1	19.21 s	12 bits

Supplementary Figures and Legends

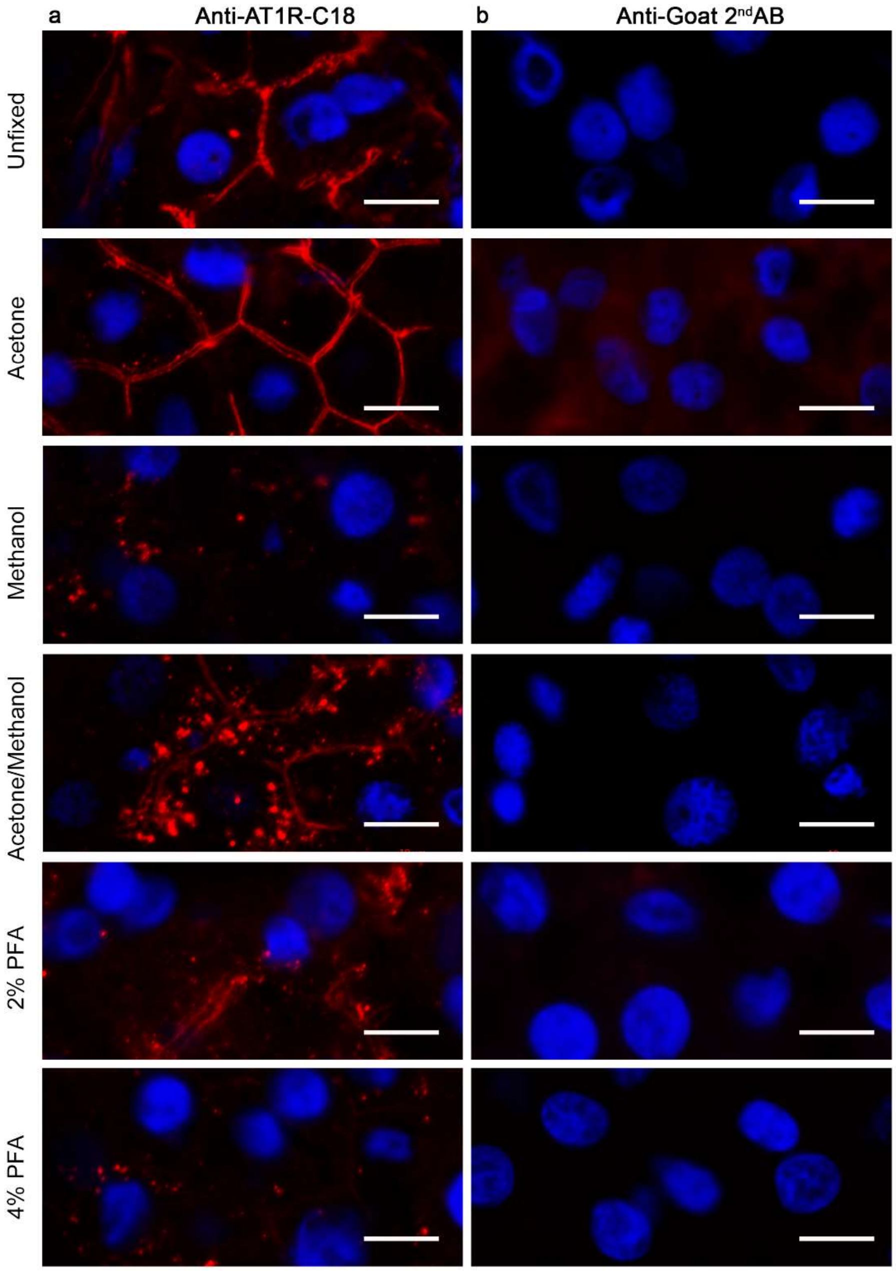

Supplementary Fig. 1 AT1R-recognition by anti-AT1R-C18 antibody in differently pre-treated human liver cryosections

(a) Specimens in the top row were air-dried, only. In the row below, the specimens were additionally treated with acetone. In the third row, acetone was replaced by methanol and in

row four by an acetone/methanol mixture (1:1). The last two rows show results obtained with 2% and 4% of PFA, respectively. The tram-track-like pattern of bile canaliculi is only visible when specimens were either air-dried or acetone treated. In (b), analogously treated specimens incubated with secondary antibody only. Confocal microscope: Zeiss LSM880. Scale bars: 10 μm (a, b).

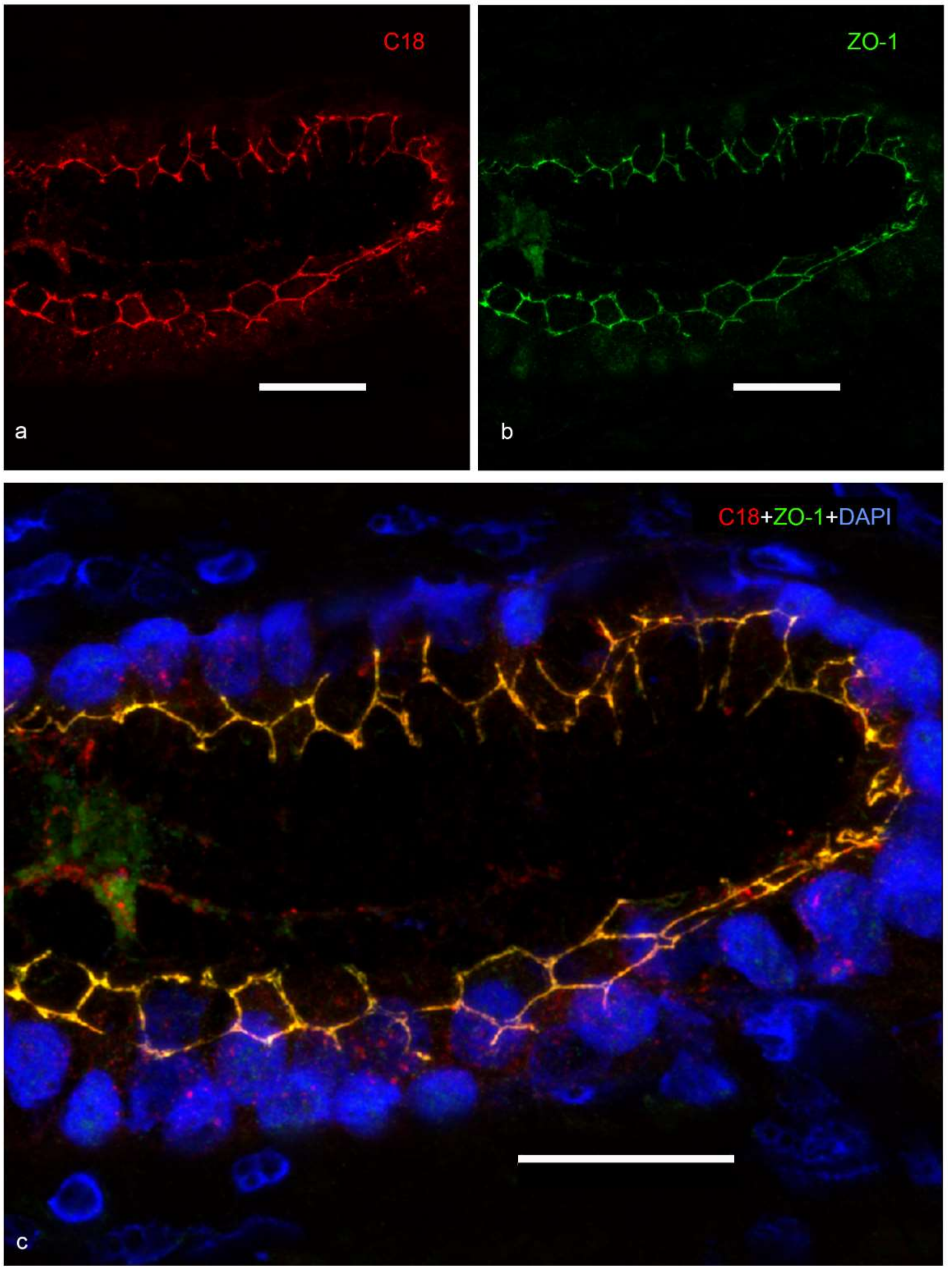

Supplementary Fig. 2 AT1R distribution in porcine bile ducts

Air-dried, acetone-treated porcine liver cryosections, permeabilized with Triton X-100 plus Tween-20 were incubated with anti-AT1R-C18 (*red*), anti-ZO-1 (*green*) and DAPI (*blue*, nuclei). (a) and (b) show images taken with single channels for AT1R-C18 and ZO-1, respectively. In (c) the single images are merged and colocalization of AT1R and ZO-1 is shown

by an almost complete signal overlap (*yellow*). Pictures were taken on Leica DMI 6000B confocal microscope. Scale bars: 20 µm (a-c).

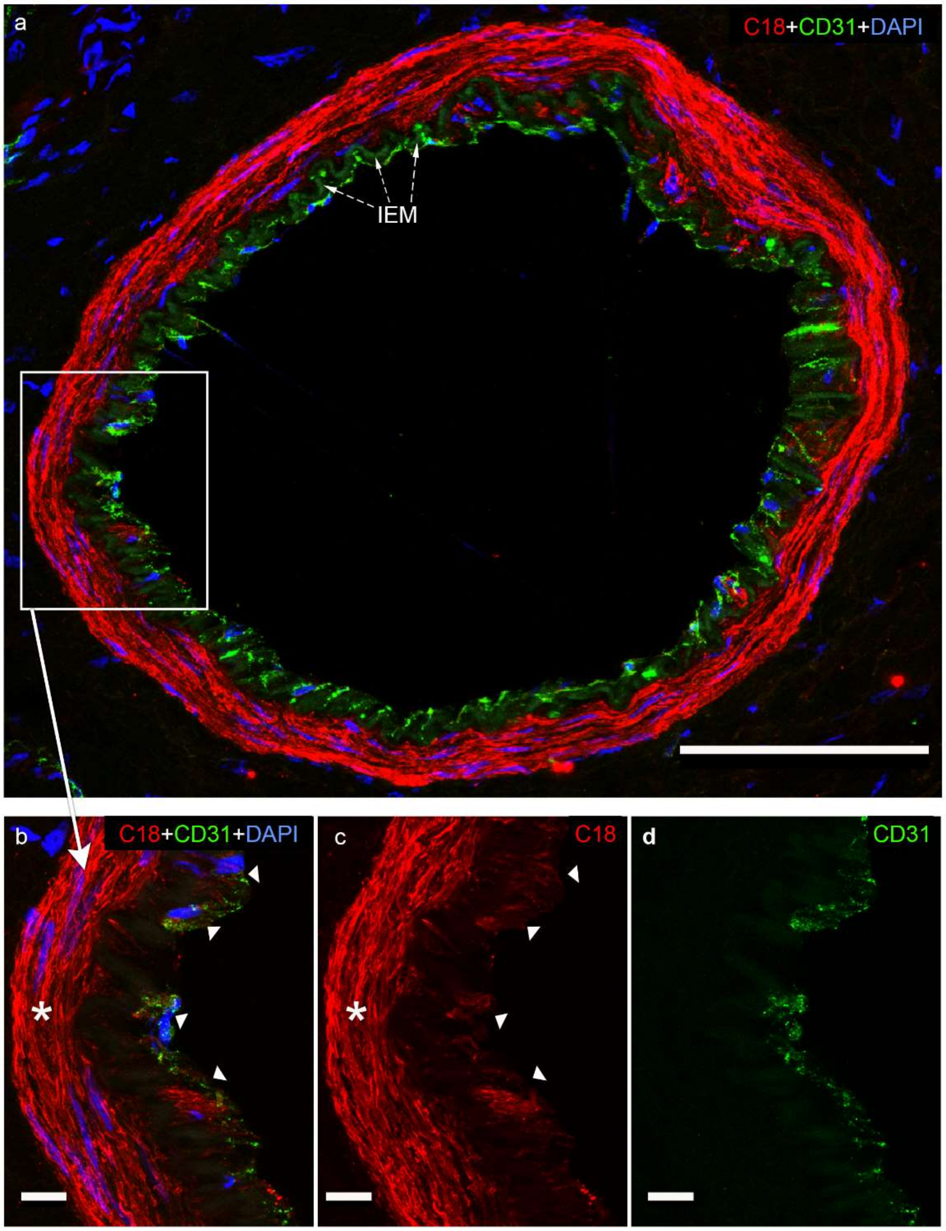

Supplementary Fig. 3 AT1R localizes predominantly in smooth muscle cells of hepatic artery

Human liver cryosections, air-dried, acetone-treated, and permeabilized were incubated with anti-AT1R-C18 antibody (*red*), endothelial cell marker anti-CD31 antibody (*green*), and DAPI (*blue*, nuclei). (a) Overview of the artery. Autofluorescence of the internal elastic membrane (IEM) of arteries was monitored in the 488 nm-channel. (b-d) Enlarged view of the boxed region in (a) using merged signals (b) of DAPI and separate fluorescence channels for anti-

AT1R-C18 antibody (c) and anti-CD31 antibody (d). (b, c) Asterisk marks tunica media; arrowheads point to CD-31-positive endothelial cells. Confocal microscope: Zeiss LSM880. Scale bars: 100 µm (a), 10 µm (b-d).

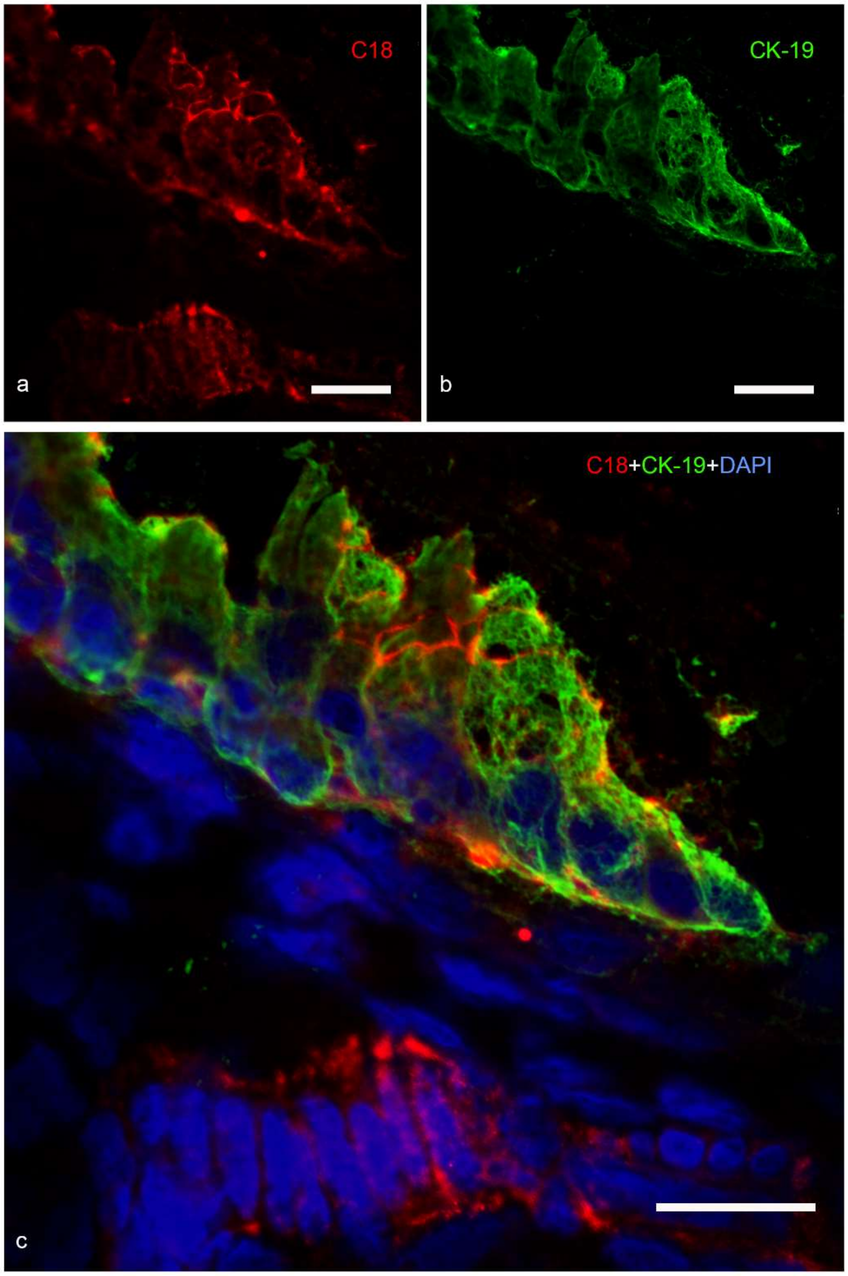

Supplementary Fig. 4 AT1R localizes to GBECs

Porcine gall bladder sections, air-dried, acetone-treated, and permeabilized were incubated with antibodies anti-AT1R-C18 (*red*), anti-CK-19 (*green*), and DAPI (*blue*, nuclei). Both AT1R and CK-19 are expressed in high prismatic GBECs. Images were taken on Zeiss LSM880 confocal microscope. Scale bars: 20 µm (a-c).

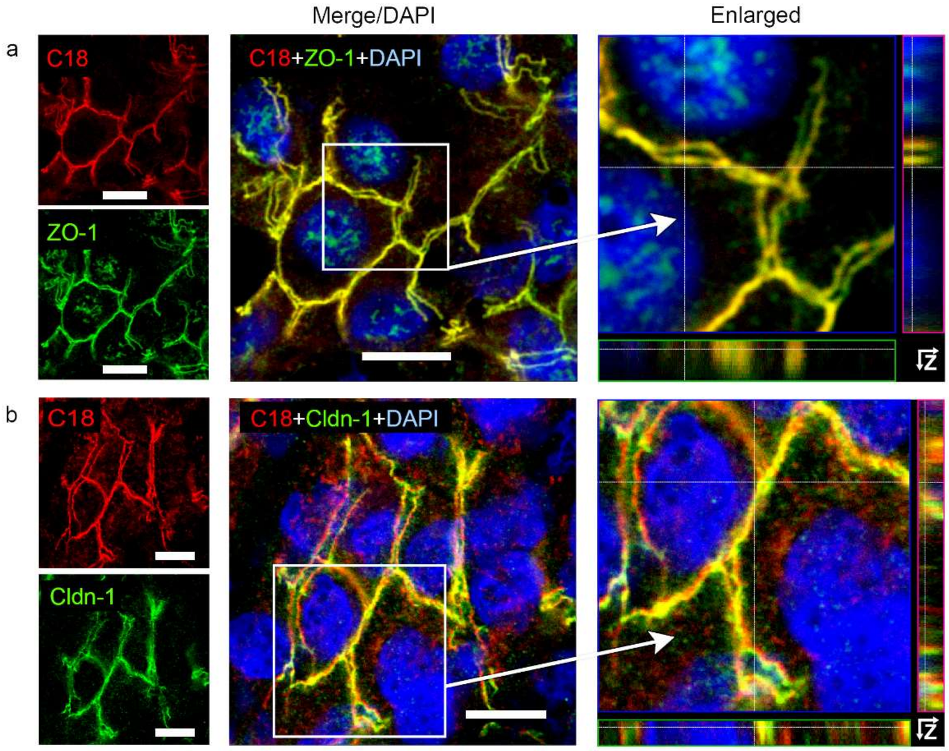

Supplementary Fig. 5 AT1R does not localize in-between the tracks of TJ proteins

Porcine cryosections, air-dried, acetone-treated, and permeabilized were incubated with anti-AT1R-C18 antibody (*red*), DAPI (*blue*, nuclei), and with antibodies anti-ZO-1 (a), or anti-claudin-1 (Cldn-1) (b). TJ proteins are in *green*. The right panel shows enlarged pictures of the boxed regions and Z-stack analyses of the maximum intensity projections. All images were taken on Leica DMI 6000B confocal microscope. Scale bars: 10 µm (a, b).

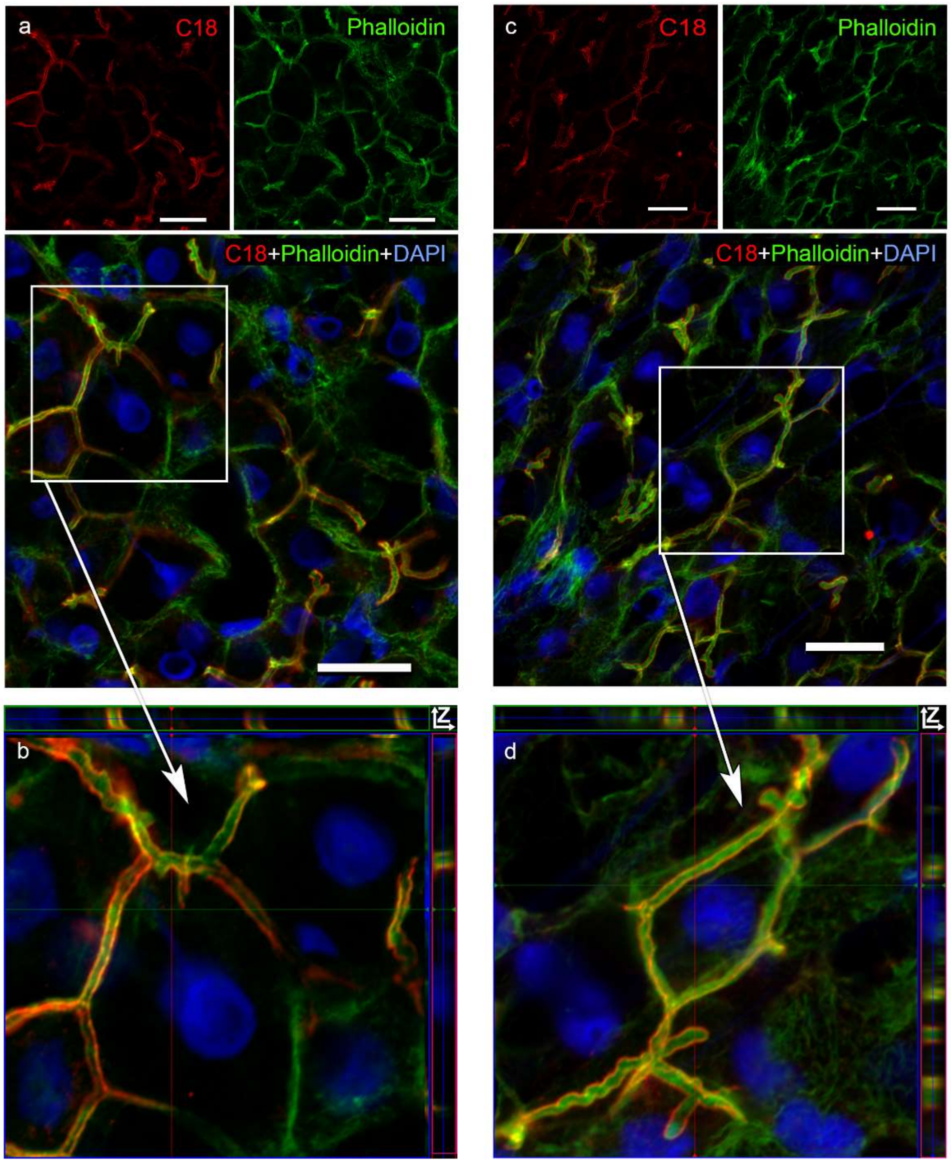

Supplementary Fig. 6 AT1R locates in close vicinity to F-actin at the apical site of human and porcine hepatocytes.

Human (a, b) and porcine (c, d) liver cryosections were incubated with anti-AT1R-C18 (red), Phalloidin-iFluor 488 (green), and DAPI (blue, nuclei). The upper row of (a) and (c) shows single channel pictures of anti-AT1R antibody and Phalloidin-iFluor 488. Signal overlap of both fluorophores appears in yellow (lower row, merged pictures). Boxed regions are enlarged in (b) and (d). Z-Stack analysis: right and bottom side of (b) and top and right side of (d). Confocal microscope: Zeiss LSM880. Scale bars: 20 µm (a, c).

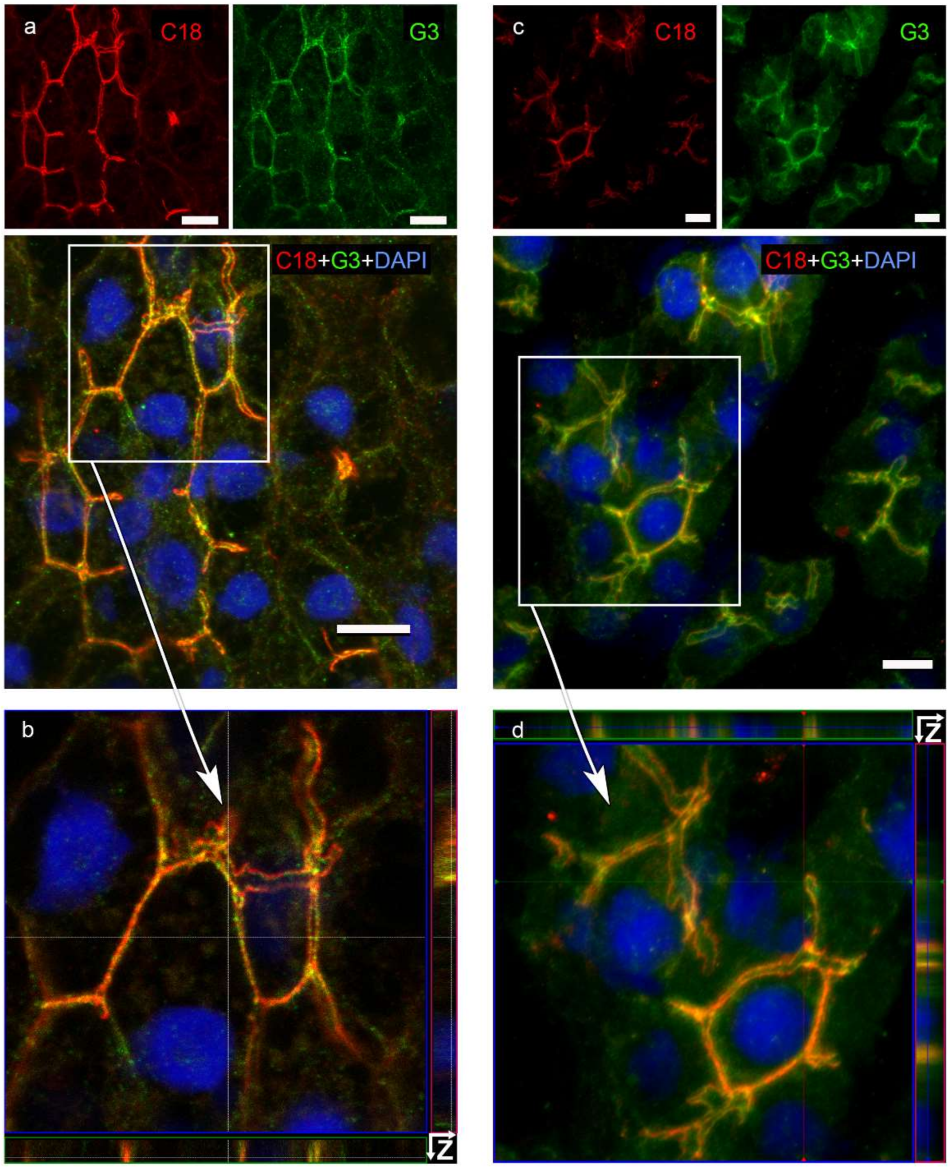

Supplementary Fig. 7 Anti-AT1R-C18 and anti-AT1R-G3 antibodies produce congruent tram-track-like pattern in hepatocytes

Human (a, b) and porcine (c, d) liver cryosections were incubated with anti-AT1R-C18 (*red*), anti-AT1R-G3 (green), and DAPI (*blue*, nuclei). The upper row of (a) and (c) shows single channel pictures of both anti-AT1R antibodies. Signal overlap of both antibodies appears in *yellow* (lower row, merged pictures). Boxed regions are enlarged in (b) and (d). Z-Stack analysis: right and bottom side of (b) and top and right side of (d). Confocal microscopes: Leica DMI 6000B for (a, b); Zeiss LSM880, for (c, d). Scale bars: 10 µm (a, c).

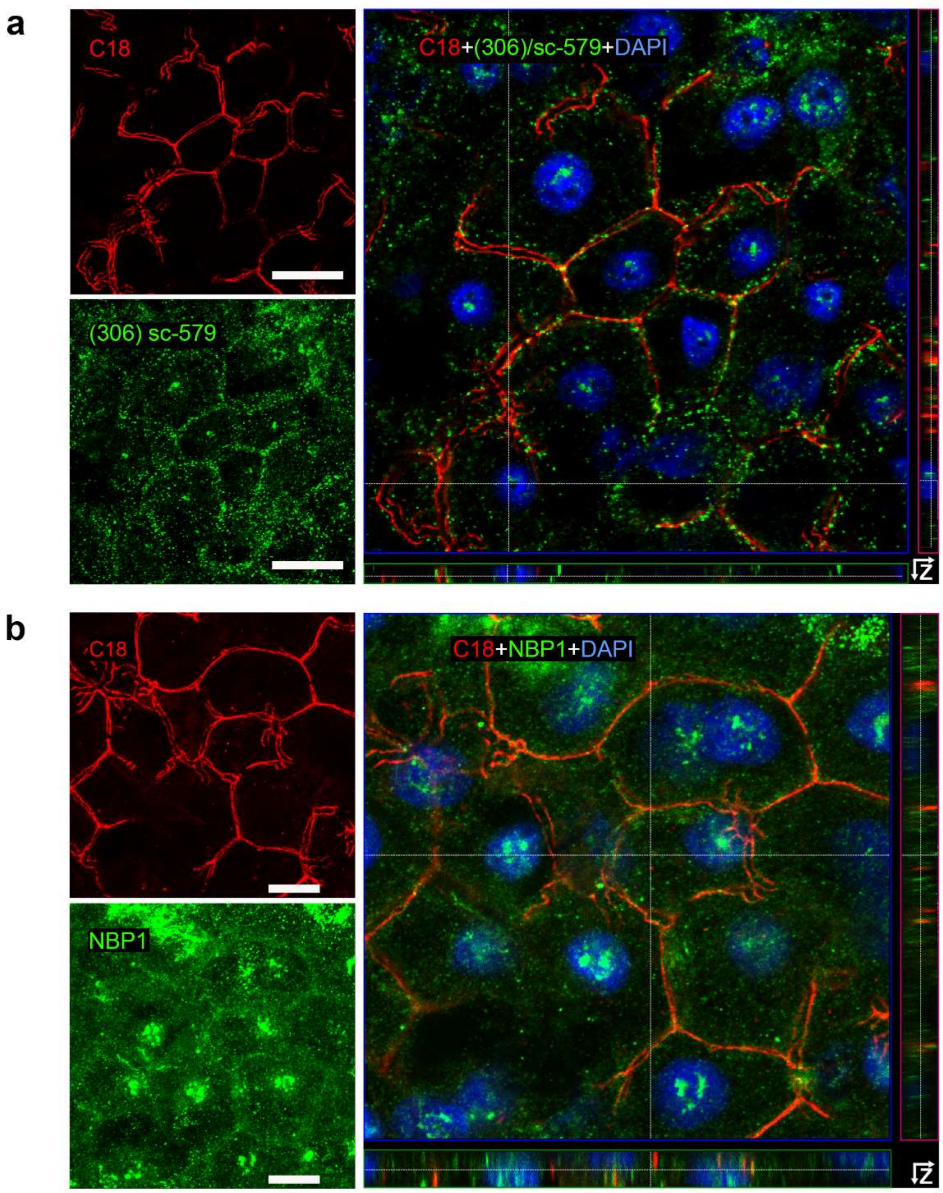

Supplementary Fig. 8 Tram-track-like distribution of AT1R in human hepatocytes detected by anti-AT1R-C18 antibody in comparison to detection by other anti-AT1R antibodies

Simultaneous incubation of human air-dried, acetone-treated, and permeabilized liver cryosections with anti-AT1R-C18 antibody (*red*), DAPI (*blue*), and either anti-AT1R-(306) sc-579 (a) or anti-AT1R-NBP1-70997 (b) antibodies, both in *green*. Signal of anti-AT1R-(306) sc-579 antibody was in close proximity to tram-tracks labeled by anti-AT1R-C18 antibody, although only sporadic overlap (*yellow*) is observed (Z-stack analysis, right). Signal of anti-

AT1R-NBP1-70997 antibody was predominantly localized to nuclei of hepatocytes and failed to reproduce the tram track-like appearance of AT1R. Confocal microscope: Leica DMI 6000B. Scale bars: 10 μm (a, b).

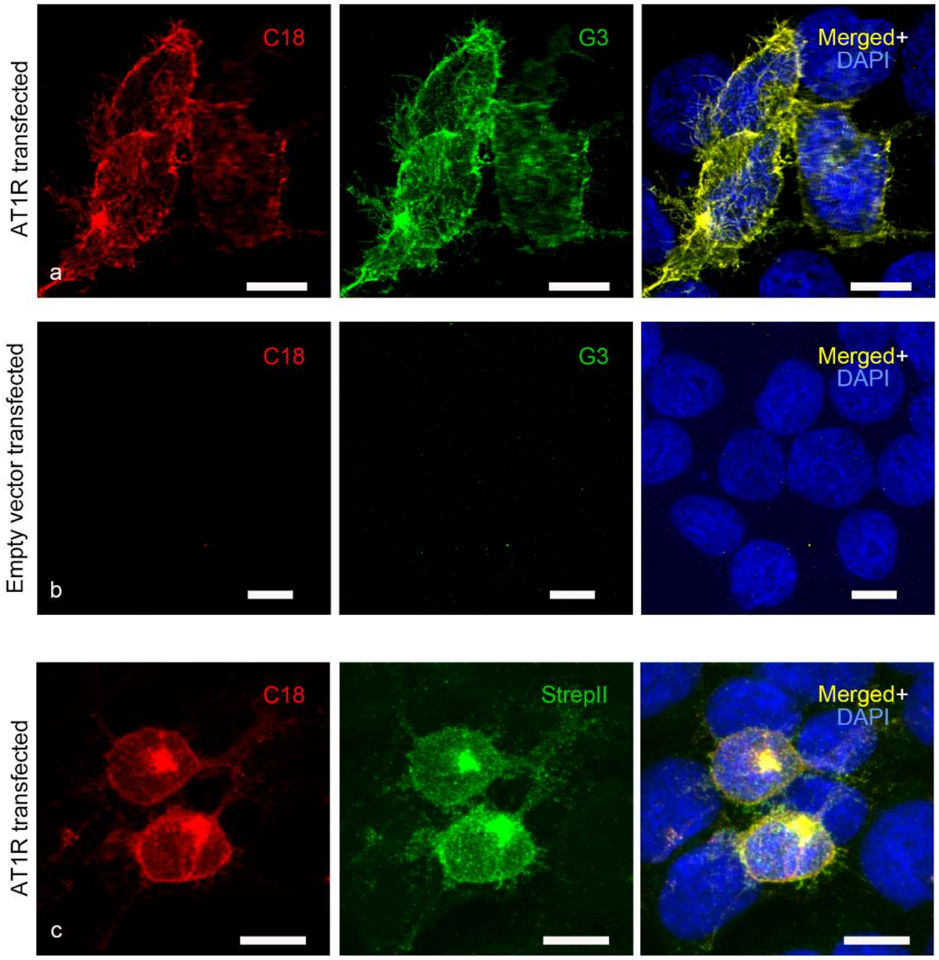

Supplementary Fig. 9 Anti-AT1R-C18, anti-AT1R-G3 and anti-Strep-tag® II antibodies provided an identical detection pattern of hhAT1R in HEK293-EBNA cells

Air-dried, acetone-treated and permeabilized hhAT1R-expressing (a, c) or empty vector-transfected HEK293-EBNA cells (b) were incubated with anti-AT1R-C18 (red), anti-AT1R-G3 (a, b, green), or anti-Strep-tag® II antibodies (c, green). Nuclei were stained with DAPI (blue). All pictures were taken on Leica DMI 6000B confocal microscope. Scale bars: 10 µm (a-c).

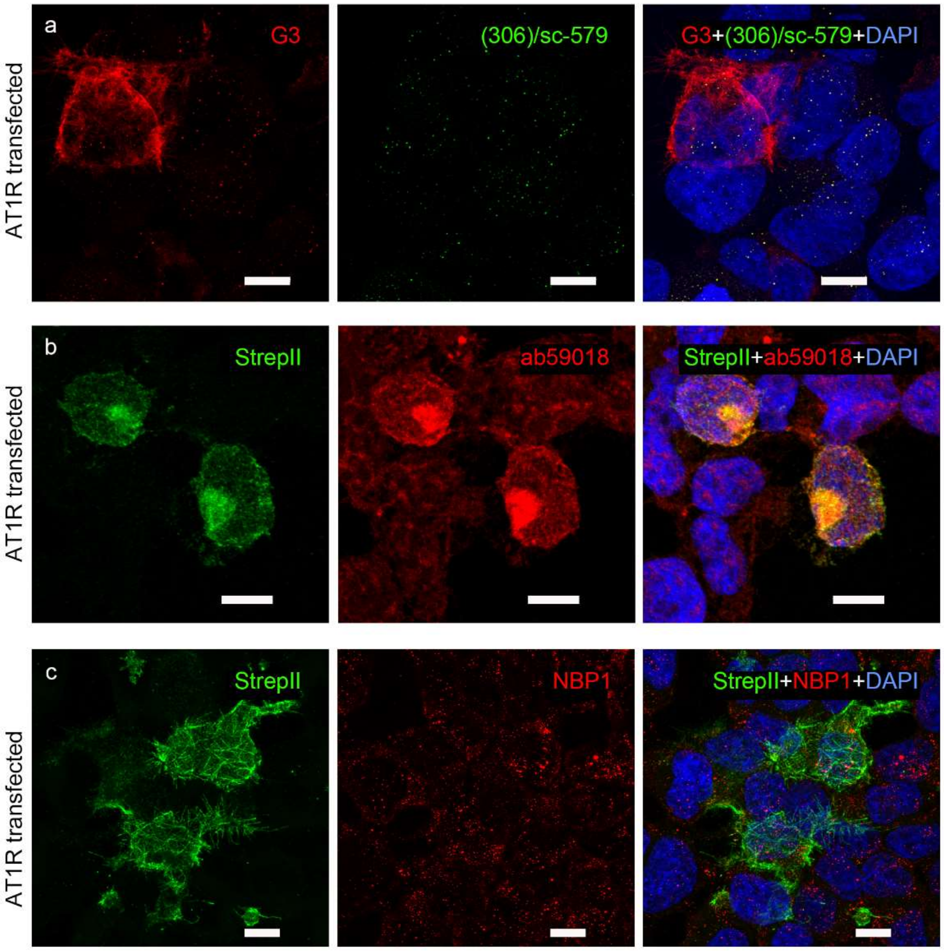

Supplementary Fig. 10 Anti-AT1R-ab59018 and anti-Strep-tag® II antibodies produced congruent hhAT1R-signals in HEK293-EBNA cells

Air-dried, acetone-treated and permeabilized hhAT1R- expressing HEK293-EBNA cells were incubated with anti-AT1R antibodies as follows: (a) anti-AT1R-G3 (*red*), anti-AT1R-(306)/sc-579 (*green*); (b) anti-AT1R-ab59018 (*red*); (c) anti-AT1R-NBP1-70997 (*red*). In (b) and (c) anti-Strep-tag® II antibody (*green*) was additionally employed. All nuclei were stained with DAPI (*blue*). Signal overlap in *yellow*. All pictures were taken on Leica DMI 6000B confocal microscope. Scale bars: 10 µm (a-c).

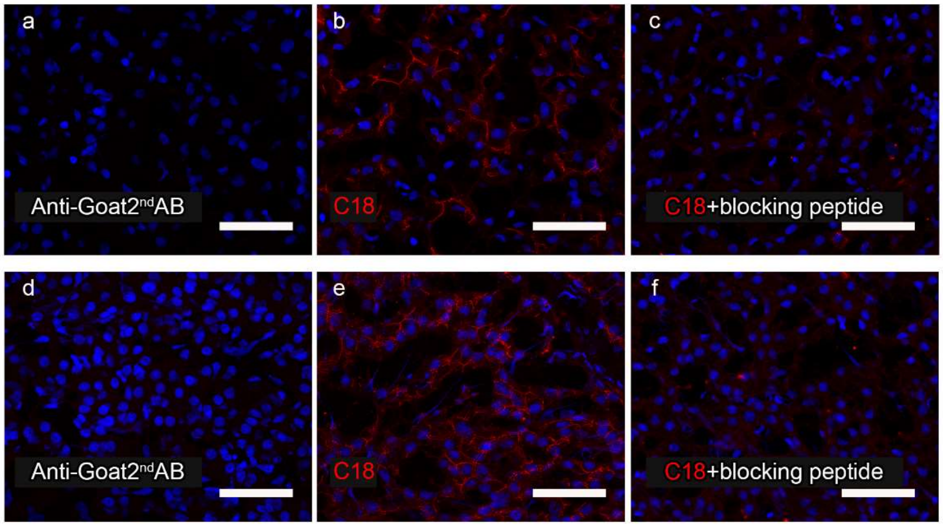

Supplementary Fig. 11 Signal loss of anti-AT1R-C18 antibody after pre-adsorption with AT1R-specific blocking peptide

Human (a-c) and porcine (d-f) liver cryosections, air-dried, acetone-treated, and permeabilized were incubated with secondary antibody only (a, d), anti-AT1R-C18 antibody (b, e), and anti-AT1R-C18 antibody pre-adsorbed with blocking peptide 1:1 (w/w) (sc-31181P) (c, f). Pictures were taken on a fluorescence microscope Leica DMI 6000B, objective 63× oil. Scale bars: 50 µm (a-f).

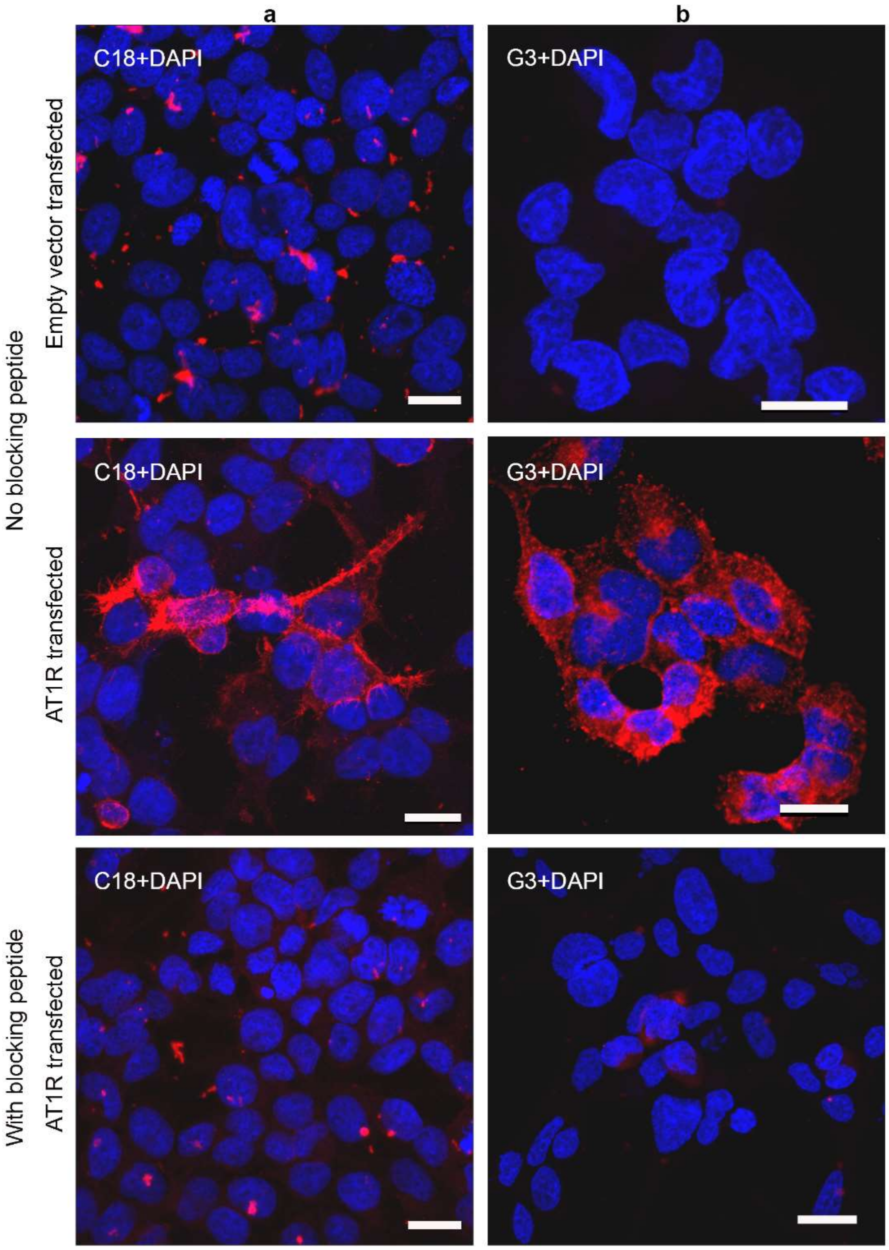

Supplementary Fig. 12 Signal loss after blocking of both anti-AT1R-C18 and anti-AT1R-G3 antibodies by an AT1R-specific peptide

Control cells (upper panel) and hhAT1R-expressing HEK293-EBNA cells (middle and bottom panel) were incubated with anti-AT1R-C18 (a), anti-AT1R-G3 (b) antibodies, and DAPI (both). Pre-adsorption of the antibodies prior incubation with an AT1R-specific commercial blocking

peptide 1:1 (w/w) (sc-31181P) (bottom panel). Pictures were taken on Zeiss LSM880 confocal microscope. Scale bars: 20 μm (a, b).

a

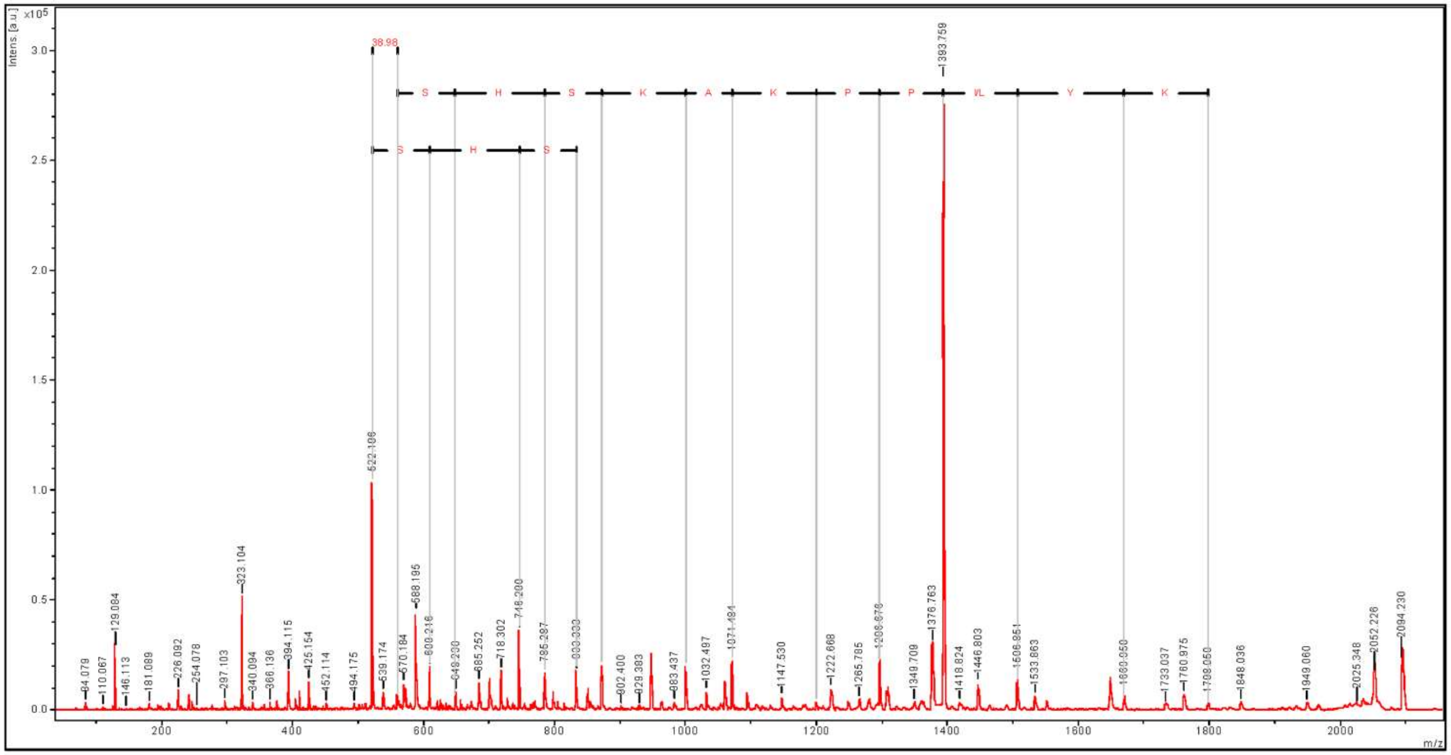

b

```
XP_003132517.1     301 FYGFLGKKFKKYFLQLLKYIPPKAKSHSSLSTKMSTLSYRPSENGSSSTKKSAPCTEVE 359
AAH22447.1         301 FYGFLGKKFKRYFLQLLKYIPPKAKSHSNLSTKMSTLSYRHSDNVSSSTKKPAPCFEVE 359
NP_114438.3        301 FYGFLGKKFKRYFLQLLKYIPPKAKSHSNLSTKMSTLSYRPSDNVSSSTKKPAPCFEVE 359
AAH68494.1         301 FYGFLGKKFKRYFLQLLKYIPPKAKSHSNLSTKMSTLSYRPSDNVSSSTKKPAPCFEVE 359
                       **********:*****************.***********  *:* ****** *** ***
```

Supplementary Fig. 13 MALDI-TOF fingerprint analysis locates blocking peptide to the C-terminus of human and porcine AT1R

(a) MALDI-TOF (m/z) fingerprint analysis of blocking peptide (sc-31181P, Lot C2814, Santa Cruz Biotechnology Inc., USA) revealed the core-sequence KYIPPKAKSHS. (b) Alignment of human and porcine AT1R sequences with Clustal Omega (1.2.4) located this sequence to the C-terminal region of AT1R (porcine: XP_003132517.1; human: AAH22447.1; NP_114438.3; AAH68494.1). MALDI-TOF was performed by the proteomic facility of the CECAD at the University of Cologne.

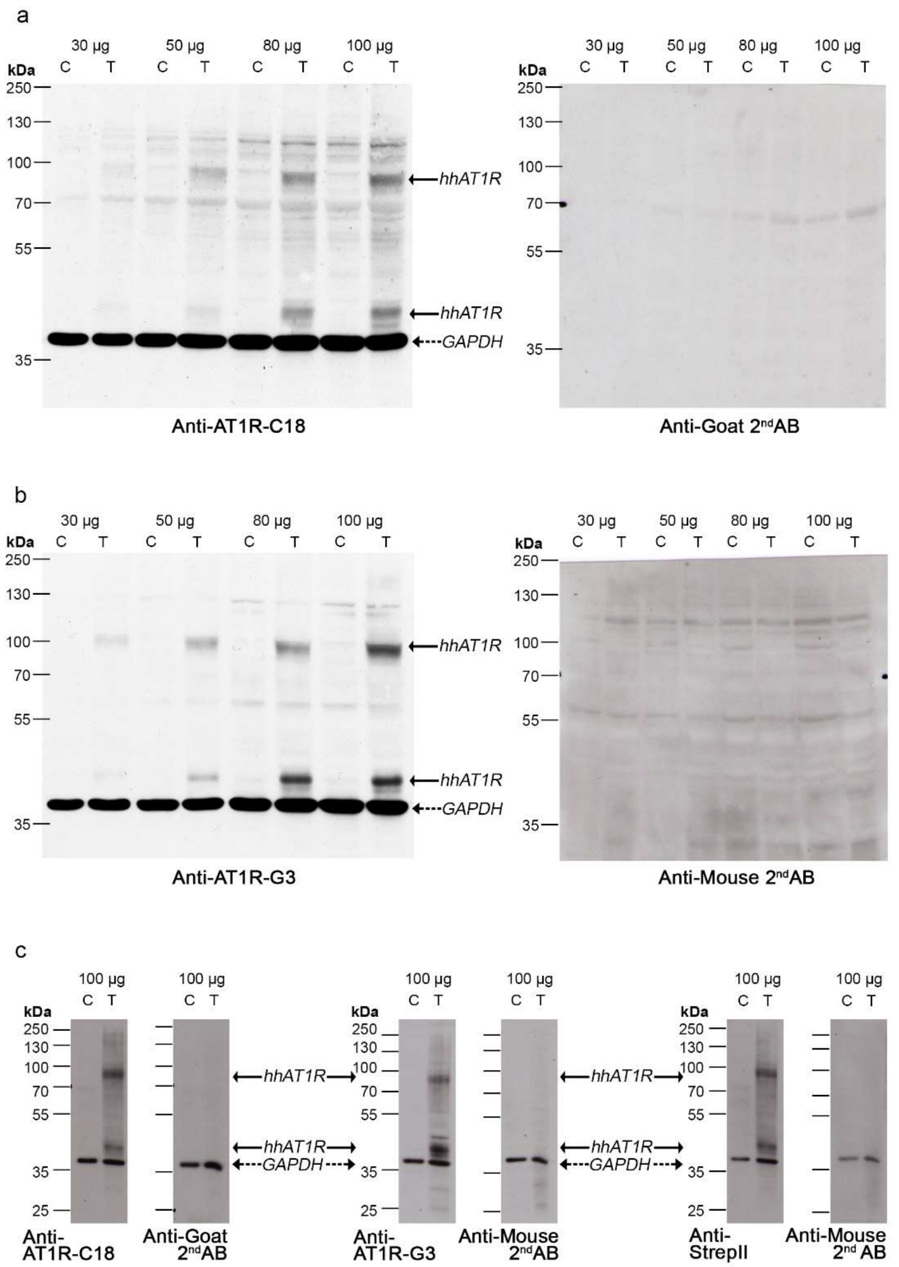

Supplementary Fig. 14 Detection of hhAT1R in Western blots

Increasing protein loads of hhAT1R-expressing HEK293-EBNA (T) and empty vector-transfected HEK293-EBNA (C) cell lysates were probed with anti-AT1R-C18 antibody (a, left) and anti-AT1R-G3 antibody (b, left), respectively. On the right side: incubation of the Western blots with their corresponding HRP-conjugated secondary antibodies, only. (c) Alternative

loaded membrane-enriched fractions of hhAT1R-expressing HEK293-EBNA and control cells were incubated with anti-AT1R-C18 (left), anti-AT1R-G3 (middle), and anti-Strep-tag® II (right) antibodies including corresponding secondary antibody controls. Arrows indicate AT1R monomers and potential dimers, arrows with dashed lines GAPDH, for normalization. Signal detection by enhanced chemiluminescence.

References

Anderson JM, Glade JL, Stevenson BR, Boyer JL, Mooseker MS (1989) Hepatic immunohistochemical localization of the tight junction protein ZO-1 in rat models of cholestasis. Am J Pathol 134:1055-1062.

Aust S, Obrist P, Jaeger W, Klimpfinger M, Tucek G, Wrba F, Penner E, Thalhammer T (2004) Subcellular localization of the ABCG2 transporter in normal and malignant human gallbladder epithelium. Lab Invest 84:1024-1036.

Barki-Harrington L, Luttrell LM, Rockman HA (2003) Dual inhibition of beta-adrenergic and angiotensin II receptors by a single antagonist: a functional role for receptor-receptor interaction in vivo. Circulation 108:1611-1618.

Benicky J, Hafko R, Sanchez-Lemus E, Aguilera G, Saavedra JM (2012) Six commercially available angiotensin II AT1 receptor antibodies are non-specific. Cell Mol Neurobiol 32:1353-1365.

Bhattacharyya D, Hammond AT, Glick BS (2010) High-quality immunofluorescence of cultured cells. Methods Mol Biol 619:403-410.

Bouressam ML, Lartaud I, Dupuis F, Lecat S (2018) No answer to the lack of specificity: mouse monoclonal antibody targeting the angiotensin II type 1 receptor AT1 fails to recognize its target. Naunyn Schmiedebergs Arch Pharmacol 391:883-889.

Durvasula RV, Petermann AT, Hiromura K, Blonski M, Pippin J, Mundel P, Pichler R, Griffin S, Couser WG, Shankland SJ (2004) Activation of a local tissue angiotensin system in podocytes by mechanical strain. Kidney Int 65:30-39.

Fox CH, Johnson FB, Whiting J, Roller PP (1985) Formaldehyde fixation. J Histochem Cytochem 33:845-853.

Fritschy JM (2008) Is my antibody-staining specific? How to deal with pitfalls of immunohistochemistry. Eur J Neurosci 28:2365-2370.

Fujita M, Furukawa H, Hattori M, Todo S, Ishida Y, Nagashima K (2000) Sequential observation of liver cell regeneration after massive hepatic necrosis in auxiliary partial orthotopic liver transplantation. Mod Pathol 13:152-157.

Gara SK, Grumati P, Urciuolo A, Bonaldo P, Kobbe B, Koch M, Paulsson M, Wagener R (2008) Three novel collagen VI chains with high homology to the alpha3 chain. J Biol Chem 283:10658-10670.

Guo S, Wang G, Yang Z (2021) Ligustilide alleviates the insulin resistance, lipid accumulation, and pathological injury with elevated phosphorylated AMPK level in rats with diabetes mellitus. J Recept Signal Transduct Res 41:85-92.

Hall PA, Stearn PM, Butler MG, D'Ardenne AJ (1987) Acetone/periodate-lysine-paraformaldehyde (PLP) fixation and improved morphology of cryostat sections for immunohistochemistry. Histopathology 11:93-101.

Hansen JL, Hansen JT, Speerschneider T, Lyngso C, Erikstrup N, Burstein ES, Weiner DM, Walther T, Makita N, Iiri T, Merten N, Kostenis E, Sheikh SP (2009) Lack of evidence for AT1R/B2R heterodimerization in COS-7, HEK293, and NIH3T3 cells: how common is the AT1R/B2R heterodimer? J Biol Chem 284:1831-1839.

Hansen JL, Theilade J, Haunso S, Sheikh SP (2004) Oligomerization of wild type and nonfunctional mutant angiotensin II type I receptors inhibits galphaq protein signaling but not ERK activation. J Biol Chem 279:24108-24115.

Helm ET, Curry SM, De Mille CM, Schweer WP, Burrough ER, Gabler NK (2020) Impact of viral disease hypophagia on pig jejunal function and integrity. PLoS One 15:e0227265.

Herrera M, Sparks MA, Alfonso-Pecchio AR, Harrison-Bernard LM, Coffman TM (2013a) Lack of specificity of commercial antibodies leads to misidentification of angiotensin type 1 receptor protein. Hypertension 61:253-258.

Herrera M, Sparks MA, Alfonso-Pecchio AR, Harrison-Bernard LM, Coffman TM (2013b) Response to lack of specificity of commercial antibodies leads to misidentification of angiotensin type-1 receptor protein. Hypertension 61:e32.

Horobin RW (1982) Histochemistry: an explanatory outline of histochemistry and biophysical staining. Gustav Fischer Verlag, Stuttgart; New York

Hughes A, Jones L (2011) Huntingtin localisation studies - a technical review. PLoS Curr 3:RRN1211.

Jung AR, Park YH, Kim GE, Kim MY, Jeon SH, Kim HY, Kim SY, Oh SH, Lee JY (2021) Stem Cell/Oxygen-Releasing Microparticle Enhances Erectile Function in a Cavernous Nerve Injury Model. Tissue Eng Part A 27:50-62.

Keon BH, Schafer S, Kuhn C, Grund C, Franke WW (1996) Symplekin, a novel type of tight junction plaque protein. J Cell Biol 134:1003-1018.

Lorincz A, Nusser Z (2008) Specificity of immunoreactions: the importance of testing specificity in each method. J Neurosci 28:9083-9086.

Lund R, Leth-Larsen R, Jensen ON, Ditzel HJ (2009) Efficient isolation and quantitative proteomic analysis of cancer cell plasma membrane proteins for identification of metastasis-associated cell surface markers. J Proteome Res 8:3078-3090.

Macedo LM, de Avila RI, Pedrino GR, Colugnati DB, Valadares MC, Lima EM, Borges CL, Kitten GT, Gava E, Castro CH (2021) Effect of angiotensin II and angiotensin-(1-7) on proliferation of stem cells from human dental apical papilla. J Cell Physiol 236:366-378.

Mardones G, Gonzalez A (2003) Selective plasma membrane permeabilization by freeze-thawing and immunofluorescence epitope access to determine the topology of intracellular membrane proteins. J Immunol Methods 275:169-177.

Michel MC, Wieland T, Tsujimoto G (2009) How reliable are G-protein-coupled receptor antibodies? Naunyn Schmiedebergs Arch Pharmacol 379:385-388.

Noguchi M, Furuya S, Takeuchi T, Hirohashi S (1997) Modified formalin and methanol fixation methods for molecular biological and morphological analyses. Pathol Int 47:685-691.

Penes MC, Li X, Nagy JI (2005) Expression of zonula occludens-1 (ZO-1) and the transcription factor ZO-1-associated nucleic acid-binding protein (ZONAB)-MsY3 in glial cells and colocalization at oligodendrocyte and astrocyte gap junctions in mouse brain. Eur J Neurosci 22:404-418.

Rateri DL, Moorleghen JJ, Balakrishnan A, Owens AP, 3rd, Howatt DA, Subramanian V, Poduri A, Charnigo R, Cassis LA, Daugherty A (2011) Endothelial cell-specific deficiency of Ang II type 1a receptors attenuates Ang II-induced ascending aortic aneurysms in LDL receptor-/- mice. Circ Res 108:574-581.

Scalia CR, Boi G, Bolognesi MM, Riva L, Manzoni M, DeSmedt L, Bosisio FM, Ronchi S, Leone BE, Cattoretti G (2017) Antigen Masking During Fixation and Embedding, Dissected. J Histochem Cytochem 65:5-20.

Schafer S, Stumpp S, Franke WW (1996) Immunological identification and characterization of the desmosomal cadherin Dsg2 in coupled and uncoupled epithelial cells and in human tissues. Differentiation 60:99-108.

Schneider MR, Hiltwein F, Grill J, Blum H, Krebs S, Klanner A, Bauersachs S, Bruns C, Longerich T, Horst D, Brandl L, de Toni E, Herbst A, Kolligs FT (2014) Evidence for a role of E-cadherin in suppressing liver carcinogenesis in mice and men. Carcinogenesis 35:1855-1862.

Sharma NM, Zheng H, Li YF, Patel KP (2012) Nitric oxide inhibits the expression of AT1 receptors in neurons. Am J Physiol Cell Physiol 302:C1162-1173.

Shin MY, Yong CC, Oh S (2020) Regulatory Effect of Lactobacillus brevis Bmb6 on Gut Barrier Functions in Experimental Colitis. Foods 9.

Tang JM, Shi N, Dong K, Brown SA, Coleman AE, Boegehold MA, Chen SY (2018) Response Gene to Complement 32 Maintains Blood Pressure Homeostasis by Regulating alpha-Adrenergic Receptor Expression. Circ Res 123:1080-1090.

Tran T, Sundaram CP, Bahler CD, Eble JN, Grignon DJ, Monn MF, Simper NB, Cheng L (2015) Correcting the Shrinkage Effects of Formalin Fixation and Tissue Processing for Renal Tumors: toward Standardization of Pathological Reporting of Tumor Size. J Cancer 6:759-766.

Young BM, Nguyen E, Chedrawe MAJ, Rainey JK, Dupre DJ (2017) Differential Contribution of Transmembrane Domains IV, V, VI, and VII to Human Angiotensin II Type 1 Receptor Homomer Formation. J Biol Chem 292:3341-3350.
